# Longitudinal multi-omics analysis uncovers the altered landscape of gut microbiota and plasma metabolome in response to high altitude

**DOI:** 10.1186/s40168-024-01781-5

**Published:** 2024-04-05

**Authors:** Yang Han, Xiaoshuang Liu, Qian Jia, Jiayu Xu, Jinlong Shi, Xiang Li, Guotong Xie, Xiaojing Zhao, Kunlun He

**Affiliations:** 1 Medical Big Data Research Center, Medical Innovation Research Division, Chinese PLA General Hospital, Beijing, China; 2 Beijing Key Laboratory of Precision Medicine for Chronic Heart Failure, Medical Innovation Research Division, Chinese PLA General Hospital, Beijing, China; 3 National Engineering Research Center for Medical Big Data Application Technology, Chinese PLA General Hospital, Beijing, China; 4 Ping An Healthcare Technology, Beijing, China; 5 Ping An Healthcare Technology, Ping An Health Cloud Company Limited, Beijing, China

**Keywords:** Gut microbiota, Altitude, Hypoxia, Metabolome, Purine metabolism

## Abstract

**Background:**

Gut microbiota is significantly influenced by altitude. However, the dynamics of gut microbiota in relation to altitude remains undisclosed.

**Methods:**

In this study, we investigated the microbiome profile of 610 healthy young men from three different places in China, grouped by altitude, duration of residence, and ethnicity. We conducted widely targeted metabolomic profiling and clinical testing to explore metabolic characteristics.

**Results:**

Our findings revealed that as the Han individuals migrated from low altitude to high latitude, the gut microbiota gradually converged towards that of the Tibetan populations but reversed upon returning to lower altitude. Across different cohorts, we identified 51 species specifically enriched during acclimatization and 57 species enriched during deacclimatization to high altitude. Notably, *Prevotella copri* was found to be the most enriched taxon in both Tibetan and Han populations after ascending to high altitude. Furthermore, significant variations in host plasma metabolome and clinical indices at high altitude could be largely explained by changes in gut microbiota composition. Similar to Tibetans, 41 plasma metabolites, such as lactic acid, sphingosine-1-phosphate, taurine, and inositol, were significantly elevated in Han populations after ascending to high altitude. Germ-free animal experiments demonstrated that certain species, such as *Escherichia coli* and *Klebsiella pneumoniae*, which exhibited altitude-dependent variations in human populations, might play crucial roles in host purine metabolism.

**Conclusions:**

This study provides insights into the dynamics of gut microbiota and host plasma metabolome with respect to altitude changes, indicating that their dynamics may have implications for host health at high altitude and contribute to host adaptation.

Video Abstract

**Supplementary Information:**

The online version contains supplementary material available at 10.1186/s40168-024-01781-5.

## Introduction

Living at high altitude presents various challenges for humans, but millions of lowlanders seek out the experience each year as hikers, skiers, or mountaineers. The Qinghai-Tibet Plateau (Tibetan Plateau) in China is a representative high-altitude environment with an average altitude of 4500 meters (m). It is mainly characterized by decreased barometric pressure, hypoxia, low temperature, and intense ultraviolet light, which significantly affect human health. A previous study on the gut microbiota of Chinese Han living in the plain and Tibetan Plateau revealed that high altitude may contribute to shaping human gut microbiota [1]. Furthermore, Tibetans, as the indigenous inhabitants of the Tibetan Plateau, exhibited significant variations in their gut microbial structure with increasing altitude [2], indicating that altitude has an impact on the gut microbiota of both the Han and Tibetan.

Generally, the journey from lowland to high altitude and back to lowland can be divided into three stages: acute response, acclimatization, and deacclimatization. The acute response stage occurs within a week of arriving at high altitude and is often accompanied by acute mountain sickness (AMS), which is the most prevalent ailment experienced by lowlanders at high altitude. Symptoms of AMS typically resolve 3 months after arriving at high altitude during the acclimatization stage. Despite significant advancements in comprehending the molecular mechanisms underlying altitude acclimatization, the determinants of successful acclimatization remain poorly understood [3]. Upon descending to lower altitudes following high-altitude acclimatization, lowlanders gradually lose their hypoxia tolerance over time and encounter alterations in hemoglobin and hormone levels, known as high-altitude deacclimatization [4]. The body undergoes distinct impairments during different stages. However, it remains unclear whether dynamic gut microbial ecosystems are characterized by different stages and participate in altitude sickness.

Beyond the mere identification of gut microbial taxa associated with altitude adaptation and acclimatization, efforts have been made to elucidate the potential functions of signature microbes. Changes in the gut microbiota and metabolites contribute to the development of altitude-induced cardiac hypertrophy in rats during hypobaric hypoxia challenge [5], implying a possible link between altered gut microbes after high altitude exposure and the occurrence of altitude sickness. Therefore, it is imperative to unravel changes in gut microbiota and blood metabolites as well as their associations following ascent to high altitude.

To gain a more profound understanding of the dynamics of gut microbiota after ascending to the high altitude and address the aforementioned knowledge gaps, we conducted a comprehensive longitudinal study integrating multidimensional datasets on the gut microbiome, plasma metabolome, and clinical indices of 138 Tibetans and 472 Hans from three places in China: Kashgar Prefecture (1700 m), Hotan Prefecture (1300 m), and Ngari Prefecture (Tibetan Plateau, 4300 m). All participants were divided into six groups according to altitude, duration of residence, place, and ethnicity. This study aims to provide a comprehensive analysis of gut microbiota, plasma metabolome, and clinical indices in the context of high altitude.

## Methods

### Stool and plasma samples collection and clinical testing

The fresh stool samples were immediately frozen in liquid nitrogen and stored at − 80 °C. DNA extraction was performed using a magnetic stool DNA kit (TIANGEN, China) according to the manufacturer’s instructions, with the addition of special grinding beads to effectively lyse complex components in fecal samples. Fasting venous blood collected with EDTA-K_2_ was centrifuged at 4000 rpm for 15 min to separate plasma, which was then stored at − 80 °C. Plasma clinical indices were analyzed using a hematology analyzer (Cobas 6000; Roche, USA) at Chinese PLA General Hospital. All participants voluntarily participated in this study and provided signed informed consent forms before sample collection. This study was approved by the Ethics Committee of the Chinese PLA General Hospital and conducted in accordance with national and institutional ethical guidelines (S2020-517–01).

### Metagenomic sequencing

#### Quality control

Shotgun paired-end sequencing of all DNA samples was conducted on a HiSeq 2500 instrument (Illumina, USA), generating reads with a fragment length of 350 bp and a read length of 150 bp at Novogene Company (China). High-quality reads were obtained by filtering out reads containing low-quality bases (quality value ≤ 38), ambiguous “*N*” bases, adapter contamination, and human genome sequences from raw data using Readfq (v8.0) and Bowtie2 (v2.2.4) [6]. The human genome (GRCh37) was used as reference for decontamination. Clean reads were assembled into scaftigs (continuous sequences within scaffolds) using SOAPdenovo software (v2.04) [7] with default parameters. Unused reads were selected through mapping against scaftigs for subsequent mixed assembly using Bowtie2 under identical parameters.

#### Taxonomy and function annotation and abundance profiling

The assembled scaftigs were utilized for gene prediction using MetaGeneMark (v2.1) [8] with default parameters. CD-HIT (v4.5.8) [9] was employed to obtain a unique initial gene catalogue, which was then mapped to the clean data in order to acquire the gene catalogue (Unigenes). The unigenes were aligned to the NCBI NR (v2018.01) database using DIAMOND (v0.9.9) [10]. The alignment results were used for conducting the lowest common ancestor algorithm, enabling us to obtain taxonomic hierarchy where the abundance of a taxonomic level in one sample equaled the sum of annotated genes assigned to it. Furthermore, we aligned the gene catalogue with Kyoto Encyclopedia of Genes and Genomes (KEGG) database (v2018.01) using DIAMOND in order to acquire functional hierarchy and their relative abundance.

### Microbiota diversity comparison

Microbiota diversity analysis was conducted using R package vegan (v2.5–6). The Shannon diversity index was used to assess α-diversity, while principal coordinate analysis (PCoA) based on Bray–Curtis distance and analysis of similarity (Anosim) were conducted to evaluate differences in microbial structure and composition across groups. Hierarchical clustering analysis was performed based on the mean abundance of species in each group.

### Genome assembly

We performed genome assembly for *Alistipes sp.CAG:435* and *Alistipes sp.CAG:514* by mapping reads from each sample obtained from Tibetan4k_NP onto a reference genome. The mapped reads were assembled using SOAP denovo (v2.04), SPAdes (v3.10.0), and ABySS (v1.3.7) respectively. Subsequently, the contigs generated from these assemblies were integrated utilizing Contig Integrator for Sequence Assembly (CISA). The preliminary assembly results underwent optimization through GapCloser (v1.12). Gene prediction was carried out utilizing GeneMarkS (v4.17). The gene function analysis methods employed were consistent with those used in the functional annotation of the sequencing dataset. Based on the assembly outcomes, the results with the best quality were selected for functional enrichment analysis. Sequence alignment and gene enrichment analyses were conducted through online tool BlastKOALA.

### Widely targeted mass spectrometry

#### Plasma samples preparation for ultra-performance liquid chromatography

Plasma samples (50 μL) were transferred into Eppendorf tubes, followed by the addition of pre-chilled ice-cold methanol containing 1 µg/mL of 2-chlorophenylalanine as an internal standard. The mixture was stirred for 3 min and centrifuged at 12,000 r/min for 10 min at a temperature of 4 °C. The resulting supernatant was further centrifuged at the same speed and temperature for another 5 min, and the new supernatant was retained for ultra-performance liquid chromatography-tandem mass spectrometry (UPLC-MS/MS) analysis.

#### UPLC-MS/MS conditions

Metabolites were separated using an ACQUITY UPLC HSS T3 C18 column (1.8 µm, 2.1 × 100 mm, Waters, MA, USA). The mobile phase consisted of ultrapure water containing 0.04% acetic acid (solvent A), and acetonitrile containing 0.04% acetic acid (solvent B). Metabolites were eluted using the following gradient: at 0 min, A:B ratio was 95:5, (v/v); at 11 min, it changed to a ratio of 5:95; at 12 min, it returned to a ratio of 95:5; and finally at 14 min, it remained constant at a ratio of 95:5. The flow rate of the mobile phase was set to be 0.4 mL/min with a column temperature maintained at 40 °C and an injection volume of 2 μL. The mass spectrometry conditions were as follows: electrospray ionization temperature was set to 500 °C with positive voltage applied as 5500 V and negative voltage as − 4500 V; gas I pressure was 55 psi while gas II pressure was 60 psi; curtain gas pressure was 25 psi; high collision-activated dissociation mode was used. In triple quadrupole, each ion pair underwent scanning based on optimized declustering potential and collision energy.

#### Plasma metabolites data analysis and quality control

Mass spectrometry data were processed using Analyst 1.6.3. Qualitative and quantitative analyses of the metabolites in the samples were conducted based on the local metabolic database. To ensure repeatability, a quality control sample was prepared by mixing sample extracts and injected after every 10 analyzed samples during instrumental analysis.

### Statistical analysis and visualization

The normality test was employed to assess the conformity of the data with a normal distribution, subsequently guiding the selection between parametric and non-parametric tests. Differential abundance of phyla, genera, species, and KEGG functions was assessed using a two-tailed Wilcoxon rank sum test between groups, and the resulting *p* values were adjusted for multiple testing (*q* value) using the false discovery rate (FDR) by Benjamin-Hochberg method. Species with an abundance greater than 0.01% in at least half of the samples in any group and a *q* value less than 0.05 were retained. Principal component analysis (PCA) was conducted to evaluate differences in the composition of the plasma metabolome and clinical indices across groups. The differential analysis of plasma metabolites and clinical indices was performed using a two-tailed Wilcoxon rank sum test, with adjusted *p* values by FDR less than 0.05 considered significant. The fold change of species, metabolites, and clinical indices was calculated as the ratio of means between each group. Statistical analyses were performed in R (v4.1.0). Differential features were visualized using R package ggplot2 (v2.0.0) and Python package GraPhlAn (v0.9.7) [11].

### Machine learning

The eXtreme Gradient Boosting (XGBoost) [12] was used to classify the Han and Tibetan populations, as well as the Han population residing at different altitudes based on the abundance of gut microbes. The area under the receiver operating characteristic curve (AUC) and 95% confidence interval were used to evaluate the performance of the models. All species were first used as raw features for XGBoost for feature selection. To find the most appropriate feature number for each iteration, we fed XGBoost with different numbers of features and evaluated its performance using the mean AUC score of 10 times tenfold cross-validation. The selected features are used to construct the final classifier models. For each group of samples, 70% were randomly selected as the training dataset, and the remaining 30% were selected as the testing dataset using a stratified sampling method. Stratified sampling is a sampling method where a population is divided into similar groups called strata, and then samples are taken from each group. This method is often used to address imbalanced datasets, ensuring that the training and test datasets maintain the same distribution of class labels as the original dataset. We used the train_test_split function from the sklearn.model_selection module (v1.3.0) to conduct stratified sampling. All functions were performed using the Python package scikit-learn (v0.21.3). The significance of the AUC was estimated by a permutation test using the R package sigr (v1.1.4).

### Pathway analysis

KEGG orthology (KO) genes that were differentially abundant and involved in metabolism were annotated and displayed in the heatmap, with clustering applied to those appearing within the same module. Differentially abundant genes significantly enriched in the same KEGG module were manually constructed by modifying KEGG pathways.

### Effect size analyses

We performed an effect size analysis to determine the potential interplay between omics datasets. To assess the proportion of variance of clinical indices or metabolome explained by gut microbiome or clinical indices, firstly, adonis analysis in the R package vegan was used to estimate the “one-to-all” effect size (*R*^2^) between each single variable of the secondary omic (gut microbiome) to the whole original omic dataset (plasma metabolome) [13]. Variables with significant effects were retained (*p* < 0.05; 999 permutations). Then, the Pearson correlation coefficient between variables was calculated to eliminate redundant variables, and variables with coefficients greater than 0.4 were removed.

### Correlation analysis

Correlation analysis was performed by calculating Spearman’s rank correlation coefficient (rho) using R package psych (v2.3.3), as well as Pearson correlation coefficient (*r*) using the stat_cor function in R package ggpubr (v0.6.0). The *p* values obtained from Spearman’s rank correlation results were adjusted for multiple testing (*q* value) using FDR via Benjamin-Hochberg method. The results were visualized using R packages ggplot2, circlize [14], and cytoscape (v3.8.2) [15].

### Germ-free animal experiments and plasma uric acid test

For the animal experiments, 10-week-old germ-free C57BL/6J male mice (*n* = 15) obtained from Gempharmatech Co., Ltd., were randomly assigned to three groups: The E. coli group (*n* = 5) was gavaged with *Escherichia coli*, the K. pneu group (*n* = 5) was gavaged with *Klebsiella pneumoniae*, and the control group (*n* = 5) was gavaged with saline. Animal research was approved by the Institutional Animal Care and Use Committee (GPTAP20220429-01) of Gempharmatech Co., Ltd., and was certified by the Association for Assessment and Accreditation of Laboratory Animal Care. Animal experiments were performed strictly in accordance with the Guide for the Care and Use of Laboratory Animals published by the Chinese PLA General Hospital. Mice were gavaged with 200 µL of either *Escherichia coli* or *Klebsiella pneumoniae* (10^9^ c.f.u. mL^−1^) three times every 2 days. On the first and seventh days after the gavage experiment, fecal samples were collected to verify the results of colonization by staining microscopy with crystal violet dye. Two weeks after the gavage experiment, plasma was collected and stored at − 80 °C. Mouse plasma UA levels were detected using a mouse UA ELISA kit (BlueGene Biotech) by measuring the intensity of the color spectrophotometrically at 450 nm in a microplate reader, according to the recommended procedure.

## Results

### Study cohorts

We collected stool samples from 610 healthy young men of Tibetan and Han ethnicities from three places in China (Table 1), and the basic information of each participant is shown in Supplementary Table 1. The Han individuals residing in the plains (altitude < 1700 m) were recruited from Kashgar Prefecture (Han1k_KP) and Hotan Prefecture (Han1k_HP), while all Tibetans living on the Tibetan Plateau (altitude > 4300 m) were from Ngari Prefecture (Tibetan4k_NP). The remaining Han participants were divided into three groups based on duration of residence and altitude, forming a time-altitude cohort. That included those who ascended to the Tibetan Plateau from Kashgar Prefecture for the first time to live for 1 week (Han4k_1w, the acute response stage) and 6 months (Han4k_6m, the acclimatization stage), and those who had resided on the plateau for more than 6 months before descending back to Kashgar Prefecture for 3 months (Han4k_d3m, the deacclimatization stage).

**Table 1:** Study cohorts and group information

Groups	Metagenome analysis (*n*)	Metabolome analysis (*n*)	Clinical test (*n*)	Sampling position	Altitude (kilometers)	Residence time
Han1k_HP	182	94	95	Hotan	< 1.7	Always
Han1k_KP	50	36	38	Kashgar	< 1.7	Always
Han4k_1w	85	53	37	Ngari	> 4.3	1 week
Han4k_6m	63	53	53	Ngari	> 4.3	6 months
Han4k_d3m	92	77	77	Kashgar	< 1.7	3 months
Tibetan4k_NP	138	90	118	Ngari	> 4.3	Always

**Fig. 1:**
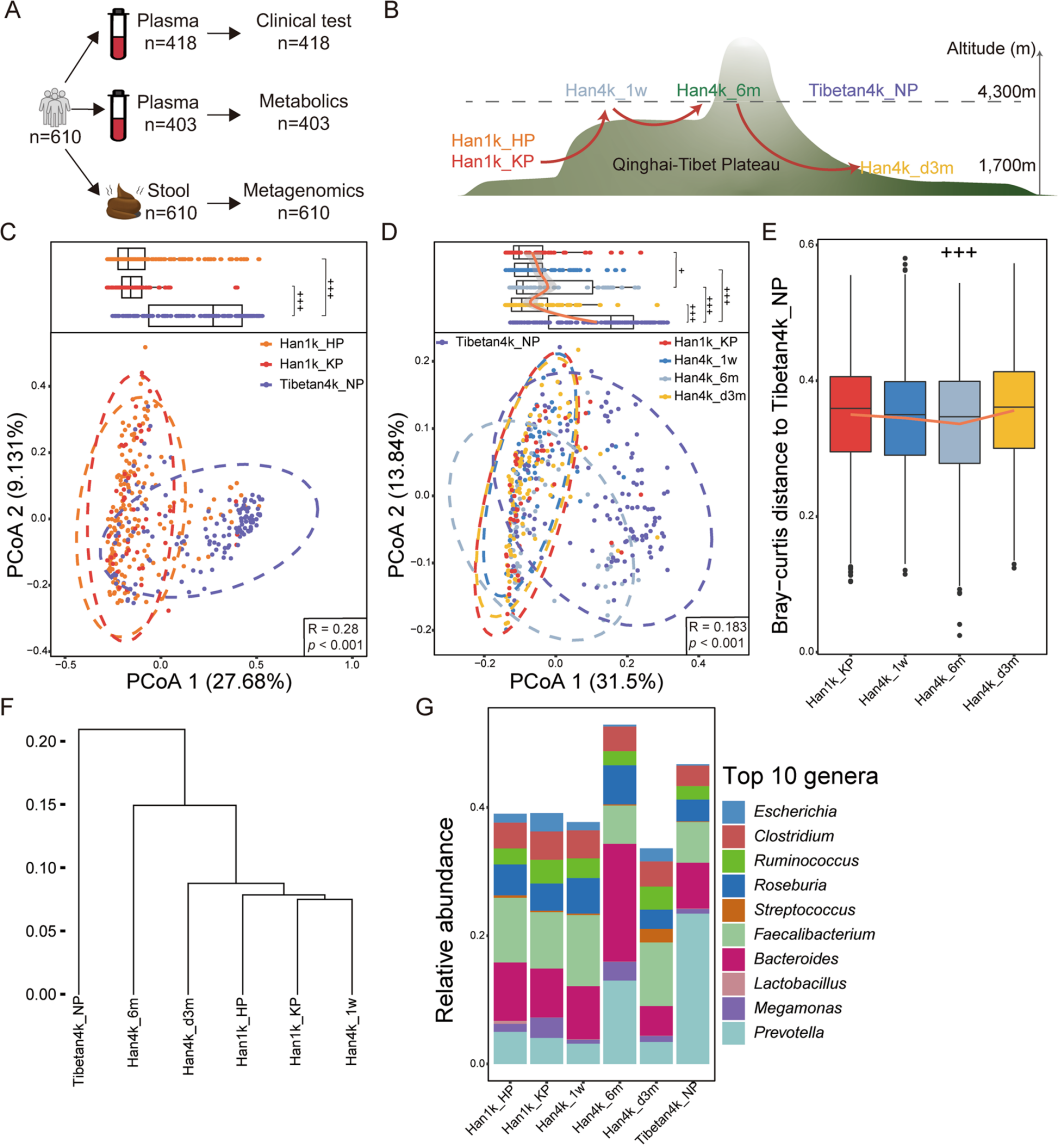
Study design and the dynamics of gut microbiota with altitude and residence time. **A** The collection of samples and the application of analytical methods. **B** The study cohort design and groups. **C** and **D** PCoA using the Bray–Curtis distance calculated based on the relative abundance of species. The 95% confidence ellipses are shown by circles. **E** The Bray–Curtis distance between the microbiota of the Han populations and Tibetan4k_NP. Wilcoxon rank sum test was used to evaluate the statistical significance of Bray–Curtis distance to Tibetan4k_NP between Han4k_6m and other groups. “ +  +  + ” above Han4k_6m indicates a significantly smaller Bray–Curtis distance for Han4k_6m compared to the other three groups, with all *q* values being less than 0.001. **F** The dendrogram for hierarchical clustering is constructed based on the Bray–Curtis distance between groups. **G** The relative abundance of top 10 genera in each group

To eliminate the confounding effects of dietary and lifestyle factors, we followed a fixed cohort of Han Chinese population as the time-altitude cohort. The diet primarily consisted of traditional Chinese cuisine, including rice, wheat, vegetables, and meat. Most of the dishes were served daily while others followed a weekly cycle. All participants ate together in the same canteen, as required by their employer, and at the same time for meals, work, and breaks. Tibetan participants were recruited from the plateau and then lived with the Han cohort together, and the same diets and lifestyles were maintained. In addition, in order to eliminate the influence of other confounding factors, participants for whom specimens were collected had to have not taken antibiotics in the 3 months before sampling. Given that alcohol is a risk factor for altitude sickness, all participants had not consumed alcoholic beverages since recruitment. The baseline characteristics of all participants are presented in Table 2. A detailed description of our study workflow can be found in Fig. 1A and B.

**Table 2:** The baseline characteristics of each group

	Han1k_HP	Han1k_KP	Han4k_1w	Han4k_6m	Han4k_d3m	Tibetan4k_NP	Significance of q value				
							Sig_1_	Sig_2_	Sig_3_	Sig_4_	Sig_5_
Age (years)	19.32 ± 1.52	19.64 ± 1.64	20.47 ± 1.52	20.17 ± 1.23	21.45 ± 1.96	19.87 ± 1.58					
BMI (kg/m^2^)	21.95 ± 2.69	21.89 ± 2.12	21.88 ± 2.36	21.91 ± 2.70	22.56 ± 2.46	21.95 ± 2.05	ns	ns	ns	ns	ns
Liver function											
TP (g/L)	71.67 ± 3.46	72.75 ± 2.93	74.11 ± 4.23	80 ± 6.32	69.65 ± 4.27	74.92 ± 8.6	ns	ns	***	***	*
BILT (μmol/L)	14.40 ± 5.40	7.81 ± 3.64	17.14 ± 5.94	17.2 ± 7.4	15.07 ± 6.02	14.42 ± 6.2	***	***	***	ns	***
ALB (g/L)	47.82 ± 2.17	48.52 ± 2.12	49.25 ± 2.8	52.67 ± 4.74	45.77 ± 2.2	48.47 ± 3.58	ns	ns	***	***	ns
ALT (U/L)	19.63 ± 12.96	18.84 ± 9.07	13.92 ± 5.86	21.87 ± 11.03	26.85 ± 16.84	26.04 ± 19.32	ns	**	ns	ns	**
AST (U/L)	23.18 ± 7.18	20.65 ± 8.55	16.5 ± 2.54	25.51 ± 6.64	22.82 ± 6.09	23.19 ± 6.48	*	**	***	**	***
GGT (U/L)	15.87 ± 7.73	14.76 ± 5.61	15.38 ± 4.45	15.51 ± 5.79	20.22 ± 22.23	23.5 ± 15.06	ns	ns	ns	ns	***
CHE (U/L)	7416 ± 1893	7719 ± 996	7825 ± 1045	8140 ± 1552	8445 ± 1110	7179 ± 1753	ns	ns	ns	ns	ns
LIPC (U/L)	20.06 ± 6.68	18.59 ± 4.98	14.44 ± 4.88	28.36 ± 11.18	28.21 ± 12.24	25.7 ± 9.07	ns	**	***	ns	***
Kidney function											
UREA (mmol/L)	5.01 ± 1.03	4.62 ± 0.76	5.09 ± 1.04	5.84 ± 1.58	5.99 ± 1.25	5.23 ± 1.14	ns	ns	***	ns	**
CREA (μmol/L)	78.37 ± 8.85	86.79 ± 9.89	85.95 ± 10.13	86.68 ± 11.78	74.19 ± 7.7	76.25 ± 11.01	***	ns	ns	***	***
UA (μmol/L)	402.60 ± 74.11	465.95 ± 85.20	385.46 ± 57.03	402 ± 65.79	408.13 ± 78.11	339.96 ± 57.61	***	***	**	ns	***
Glucose metabolism											
GLU (mmol/L)	5.25 ± 0.34	5.09 ± 0.37	3.66 ± 0.45	4.4 ± 0.61	5.06 ± 0.38	4.53 ± 0.45	*	***	***	***	***
Blood lipid											
TG (ng/mL)	8.46 ± 6.26	9.60 ± 6.52	11.67 ± 7.82	9.99 ± 7.59	10.51 ± 7.72	9.66 ± 7.47	ns	ns	ns	ns	ns
TRIGL (mmol/L)	0.95 ± 0.25	0.82 ± 0.32	0.96 ± 0.27	0.94 ± 0.31	1.05 ± 0.4	0.89 ± 0.35	**	*	*	ns	ns
HDL (mmol/L)	1.14 ± 0.20	1.17 ± 0.22	1.33 ± 0.22	1.35 ± 0.28	1.24 ± 0.27	1.26 ± 0.5	ns	*	**	*	ns
CHO (mmol/L)	3.36 ± 0.61	3.24 ± 0.58	3.69 ± 0.56	3.55 ± 0.69	3.59 ± 0.57	3.29 ± 0.63	ns	**	*	ns	ns
LDL (mmol/L)	1.98 ± 0.55	1.94 ± 0.55	2.06 ± 0.58	1.86 ± 0.58	1.97 ± 0.46	1.82 ± 0.54	ns	ns	ns	ns	ns
APOA (g/L)	1.20 ± 0.32	1.13 ± 0.13	1.36 ± 0.15	1.34 ± 0.24	1.3 ± 0.19	1.2 ± 0.18	ns	***	***	ns	ns
APOB (g/L)	0.62 ± 0.14	0.61 ± 0.15	0.65 ± 0.16	0.74 ± 0.18	0.64 ± 0.14	0.62 ± 0.16	ns	ns	**	**	ns
Myocardial enzyme											
CK (U/L)	291.6 ± 254.75	245.58 ± 203.03	131.61 ± 40.68	191.77 ± 97.29	216.47 ± 194.07	160.47 ± 63.23	ns	***	ns	ns	*
CKMB (U/L)	17.53 ± 5.44	14.23 ± 4.66	12.65 ± 3.07	17.85 ± 5.75	18.81 ± 7.69	16.35 ± 5.41	***	ns	***	ns	**
LDIP (U/L)	245.57 ± 81.34	191.32 ± 35.52	173.7 ± 31.46	288.23 ± 117.04	209.64 ± 79.82	224.64 ± 70.66	***	ns	***	***	***
Blood ion											
NH3 (μmol/L)	74.26 ± 24.57	63.16 ± 9.15	84.88 ± 34.55	73.18 ± 31.51	68.8 ± 18.03	64.74 ± 29.63	ns	***	ns	ns	ns
PHO (mmol/L)	1.17 ± 0.12	1.26 ± 0.15	0.99 ± 0.14	1.2 ± 0.17	1.11 ± 0.12	1.12 ± 0.17	**	***	*	*	***
Specific protein											
C3C (g/L)	1.06 ± 0.13	1.01 ± 0.12	1.1 ± 0.16	1.03 ± 0.16	1.05 ± 0.15	1.1 ± 0.17	*	ns	ns	ns	ns
CRPHS (mg/L)	0.77 ± 0.97	1.24 ± 1.20	0.44 ± 0.4	0.62 ± 1.58	0.79 ± 0.99	1.14 ± 1.67	**	***	***	***	ns
ProBNP (pg/mL)	19.672 ± 16.02	23.49 ± 14.98	39.97 ± 31.44	13.99 ± 11.05	19.85 ± 15.22	20.39 ± 17.16	ns	*	**	*	ns
Amylase											
AMY-L (U/L)	53.99 ± 17.67	48.89 ± 16.29	58.08 ± 18.8	67.74 ± 19.33	63.65 ± 20.77	68.25 ± 17.7	ns	*	***	ns	***
AMY-P (U/L)	22.16 ± 7.27	19.81 ± 5.37	21.36 ± 5.86	26.42 ± 6.43	26.03 ± 8.24	28.68 ± 6.26	ns	ns	***	ns	***
Tumor marker											
AFP (g/L)	1.91 ± 0.95	1.81 ± 0.78	1.63 ± 0.64	1.91 ± 0.76	2.68 ± 1.25	1.7 ± 1.19	ns	ns	ns	***	ns
CEA (ng/mL)	21.5 ± 1.19	2.12 ± 1.19	2.29 ± 1.28	2.51 ± 1.1	2.31 ± 1.23	2.31 ± 0.99	ns	ns	ns	ns	ns
CA125 (U/mL)	10.37 ± 4.24	4.82 ± 9.87	8.89 ± 3.06	11.12 ± 4.47	9.17 ± 5.66	11.43 ± 10.03	ns	ns	ns	**	ns
CA153 (U/mL)	8.10 ± 4.02	8.05 ± 3.04	7.91 ± 2.6	12.92 ± 6.09	8.91 ± 3.94	8.86 ± 4.81	ns	ns	***	***	ns
CA199 (U/mL)	8.61 ± 9.08	6.20 ± 3.26	7.41 ± 4.32	9.7 ± 7.54	9.17 ± 5.66	10.93 ± 7.21	*	ns	*	ns	***
CA724 (U/mL)	1.37 ± 1.50	3.29 ± 3.64	2.56 ± 2.79	3.64 ± 3.99	2.54 ± 2.77	1.57 ± 1.69	***	ns	ns	*	***
TPSA (ng/mL)	0.62 ± 0.35	0.57 ± 0.35	0.69 ± 0.44	0.59 ± 0.28	0.65 ± 0.31	0.59 ± 0.56	ns	ns	ns	ns	ns
FPSA (ng/mL)	0.21 ± 0.11	0.18 ± 0.08	0.25 ± 0.17	0.26 ± 0.13	0.28 ± 0.14	0.2 ± 0.14	ns	*	**	ns	ns
ProGRP (pg/mL)	33.14 ± 7.09	34.39 ± 6.25	37.33 ± 6.96	36.8 ± 12.07	35.72 ± 9.18	40.91 ± 14.06	ns	ns	ns	ns	**
CYFRA (ng/mL)	2.29 ± 0.71	2.20 ± 0.70	1.9 ± 0.69	2.29 ± 0.95	2.19 ± 0.94	2.21 ± 0.69	ns	ns	ns	ns	ns
HE4 (pmol/L)	47.56 ± 8.71	45.40 ± 7.88	46.94 ± 7.23	49.15 ± 8.62	47.5 ± 8.97	56.01 ± 9.71	ns	ns	ns	ns	***
FERR (ng/mL)	144.76 ± 79.94	128.89 ± 65.55	103.03 ± 52.58	98.41 ± 62.26	321.09 ± 237.15	210.83 ± 199.91	ns	ns	*	***	ns
Thyroid function											
T3 (nmol/L)	1.74 ± 0.25	1.78 ± 0.23	1.85 ± 0.23	1.93 ± 0.3	1.96 ± 0.31	1.92 ± 0.26	ns	ns	**	ns	**
T4 (nmol/L)	86.03 ± 14.14	86.79 ± 14.30	97.59 ± 16.05	102.49 ± 18.18	90.57 ± 14.89	87.84 ± 15.36	ns	**	***	***	ns
FT3 (pmol/L)	5.37 ± 0.49	5.13 ± 0.50	5.82 ± 0.7	5.6 ± 0.5	5.75 ± 0.5	5.66 ± 0.58	*	***	***	ns	***
FT4 (pmol/L)	17.10 ± 2.07	15.11 ± 2.15	18.76 ± 2.35	17.9 ± 2.51	16.9 ± 1.91	16.74 ± 3.67	***	***	***	*	**
TSH (mIU/L)	2.37 ± 1.38	2.16 ± 1.08	1.73 ± 0.98	2.45 ± 1.36	2.45 ± 1.39	2.69 ± 1.12	ns	ns	ns	ns	**
A-TG (IU/mL)	10.47 ± 2.86	10.19 ± 0.56	10.18 ± 0.81	11.49 ± 7.59	10.41 ± 2.26	12.09 ± 10.43	ns	ns	ns	ns	ns
A-TPO (IU/mL)	5.29 ± 0.10	5.60 ± 2.47	5.72 ± 2.68	6.29 ± 3.21	6.85 ± 7.78	5.91 ± 5.06	ns	ns	*	ns	ns
Hormone											
E2 (pmol/L)	174.06 ± 126.98	122.57 ± 35.11	155.47 ± 38.65	154.36 ± 36.43	105.52 ± 71.42	153.24 ± 73.02	***	**	***	**	**
FSH (IU/L)	4.00 ± 3.11	3.84 ± 1.54	3.29 ± 1.3	4.28 ± 1.81	3.31 ± 1.59	4.8 ± 4.11	ns	ns	ns	**	ns
LH (mIU/mL)	4.41 ± 1.57	5.32 ± 1.68	5.63 ± 2.01	4.18 ± 1.7	4.6 ± 1.54	5.46 ± 2.35	*	ns	**	ns	ns
PROG (nmol/L)	1.56 ± 0.38	34.39 ± 6.25	0.96 ± 0.42	0.75 ± 0.28	1.35 ± 0.43	1.15 ± 0.43	***	ns	***	***	ns
TESTO (nmol/L)	18.52 ± 4.40	18.14 ± 4.29	23.43 ± 5.47	26.05 ± 6.31	20.51 ± 4.41	20.22 ± 7.14	ns	***	***	***	**
CORT (nmol/L)	446.55 ± 63.61	394.96 ± 89.17	294.49 ± 121.79	368.65 ± 94.53	411.53 ± 110.03	408.72 ± 88.78	**	***	ns	*	ns
Insulin (pmol/L)	49.95 ± 18.07	39.62 ± 14.14	54.25 ± 20.08	46.89 ± 24.51	48.67 ± 23.29	42.38 ± 29.54	**	**	ns	ns	ns
CPEPTID (nmol/L)	0.58 ± 0.13	0.55 ± 0.12	0.61 ± 0.16	0.67 ± 0.17	0.57 ± 0.14	0.55 ± 0.21	ns	ns	**	**	ns
PRL (ng/mL)	17.84 ± 5.83	12.40 ± 3.66	10.72 ± 4.01	8.94 ± 2.86	12.52 ± 5.06	14.32 ± 5.38	***	ns	***	***	ns
Myocardium											
TNT-HS (ng/mL)	0.006 ± 0.002	0.004 ± 0.001	0.006 ± 0.003	0.005 ± 0.002	0.004 ± 0.002	0.005 ± 0.002	***	*	ns	ns	ns
MYO (ng/mL)	37.84 ± 15.07	28.53 ± 16.79	27.44 ± 4.65	29.06 ± 7.72	32.74 ± 32.79	29.77 ± 6.18	***	*	*	ns	***
Infectious disease											
A-HCV (COI)	0.05 ± 0.03	0.04 ± 0.03	0.05 ± 0.02	0.04 ± 0.01	0.04 ± 0.02	0.05 ± 0.03	**	***	ns	***	**
Bone metabolism											
TP1NP (ng/mL)	154.47 ± 66.14	135.10 ± 76.31	125.71 ± 74.14	125.78 ± 45.4	111.2 ± 38.98	207.16 ± 137.17	ns	ns	ns	ns	***
Septicopyemia											
IL6 (pg/mL)	1.95 ± 0.93	0.55 ± 0.12	3.26 ± 3.1	2.06 ± 1.67	2.27 ± 2.17	2.41 ± 2.0	***	***	***	ns	***
PCT (ng/mL)	0.03 ± 0.01	0.03 ± 0.01	0.03 ± 0.03	0.03 ± 0.01	0.03 ± 0.02	0.03 ± 0.02	*	ns	**	***	ns
Rheumatoid arthritis											
A-CCP (U/mL)	7.85 ± 6.33	7.00 ± 0.00	8.28 ± 6.98	9.63 ± 12.06	7.45 ± 3.51	7.5 ± 4.16	ns	ns	ns	ns	ns
Anaphylaxis											
IGE (IU/mL)	158.35 ± 212.99	153.35 ± 217.46	90.79 ± 151.39	211.36 ± 232.39	105.22 ± 135.58	87.46 ± 143.07	ns	ns	ns	*	*

*Sig*_*1*_, significance of the comparison between Han1k_HP and Han1k_KP; *Sig*_*2*_, significance of the comparison between Han1k_KP and Han4k_1w; *Sig*_*3*_, significance of the comparison between Han1k_KP and Han4k_6m; *Sig*_*4*_, significance of the comparison between Han4k_6m and Han4k_d3m; *Sig*_*5*_, significance of the comparison between Han1k_KP and Tibetan4k_NP

* indicates *q* value < 0.05

** indicates *q* value < 0.01

*** indicates *q* value < 0.001; ns indicates *q* value > 0.05

### Fluctuation of gut microbiota with altitude

Tibetans, the largest indigenous group residing in the Tibetan Plateau, have developed a range of adaptive characteristics to cope with the challenging environment. The Shannon index revealed no significant difference in terms of α-diversity between Han1k_KP and Han1k_HP, but both were lower than that of Tibetan4k_NP (Supplementary Fig. 1A). Furthermore, a principal coordinate analysis (PCoA) plot demonstrated distinct clusters among Han1k_KP, Han1k_HP, and Tibetan4k_NP over the first two dimensions (Anosim test, *R* = 0.28,* p* < 0.001, Fig. 1C), while no distinction was observed between Han1k_KP and Han1k_HP (Anosim test, *R* = 0.065, *p* = 0.062). These findings indicate a significant separation in microbial composition and structure between the Tibetan and plain-dwelling Han populations.

In the time-altitude cohort, there was no significant difference in α-diversity among Han1k_KP, Han4k_1w, and Han4k_d3m. However, compared to Han4k_1w, Han4K_6m exhibited a significantly decreased α-diversity (Supplementary Fig. 1B). PCoA results indicated that both Han4k_6m and Tibetan4k_NP showed significant dissimilarities from other groups (Anosim test, *R* = 0.183, *p* < 0.001, Fig. 1D). To further analyze similarities in microbial composition between the time-altitude cohort group and Tibetan4k_NP, the Bray–Curtis distance from other groups to Tibetan4k_NP was calculated. A clear concave pattern was observed, and Han4k_6m had the smallest community distance to Tibetan4k_NP (*p* < 0.001, Fig. 1E). Hierarchical cluster analysis also revealed that Han4k_6m was the closest group to Tibetan4k_NP, followed by Han4k_d3m (Fig. 1F). These results suggest that after ascending to plateau, the microbial composition of Hans gradually converged towards that of Tibetans over time, and it retained some plateau-specific characteristics even after descending back to plains for 3 months.

Among the dominant phyla, Bacteroidetes, Chlamydiae, and Euryarchaeota were significantly enriched in Tibetan4k_NP compared to Han1k_KP and Han1k_HP. Conversely, Firmicutes, Proteobacteria, Actinobacteria, and Verrucomicrobia displayed reversed patterns (Supplementary Table 2). A higher abundance of Bacteroidetes and a lower abundance of Firmicutes in the Tibetan have been previously reported [2]. The analysis of the top 10 most abundant genera (Fig. 1G, Supplementary Table 3) revealed that only *Prevotella*, a diverse genus of gram-negative anaerobic bacteria from Bacteroidetes and known for its proficiency in producing short-chain fatty acid (SCFA) propionate [16], was significantly enriched in Tibetan4k_NP compared to Han1k_KP and Han1k_HP. On the other hand, *Escherichia*, *Streptococcus*, and *Faecalibacterium* showed contrasting trends. In the time-altitude cohort, it was observed that *Prevotella* and *Bacteroides* showed a significant increase in abundance in Han4k_6m compared to Han1k_KP. This suggests that the increased *Prevotella* is the dominant taxon leading the gut microbiota of Han4k_6m to be similar to that of the Tibetan populations.

### Specific microbial signatures of acute response, acclimatization, and deacclimatization stages

**Fig. 2:**
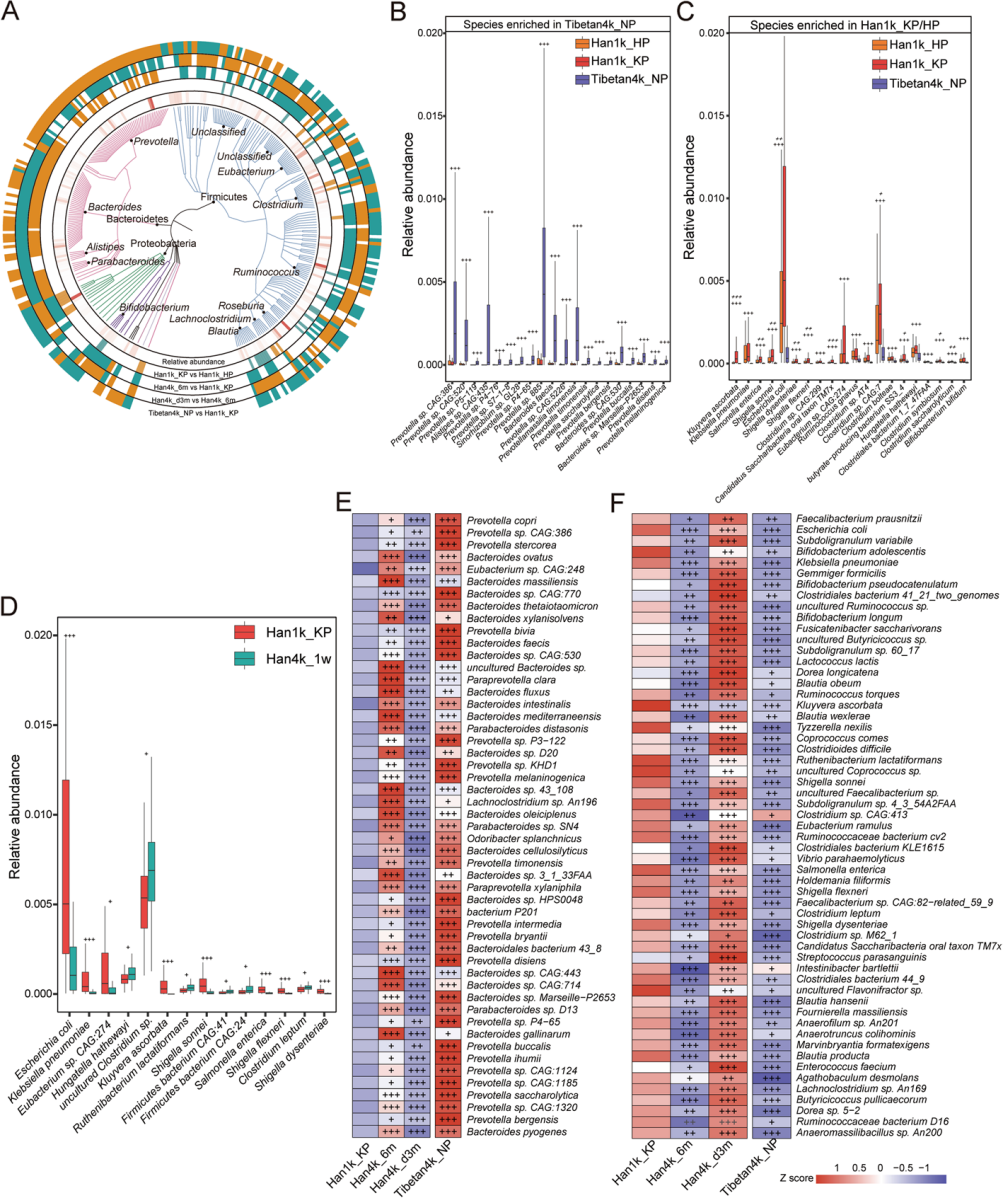
Distinct taxonomic signatures associated with the acute response, acclimatization, and deacclimatization. **A** In total, 296 differentially abundant species are displayed in a phylogenetic tree, primarily clustered within the phyla Firmicutes, Bacteroidetes, and Proteobacteria. The outer circles indicate species that are significantly enriched (orange) or depleted (green) in the group before “vs.” The innermost circle shows the average relative abundance of species across all samples. **B** The differentially enriched species in Tibetan4k_NP compared to Han1k_KP with the top 20 significance levels were displayed. The statistical significance of Tibetan4k_NP compared to both Han1k_KP and Han1k_HP is indicated above the box plots using regular plus signs. **C** The differentially enriched species in both Han1k_KP and Han1k_HP compared to Tibetan4k_NP with top 20 significance. The statistical significance of Han1k_KP and Han1k_HP compared to Tibetan4k_NP is indicated above the box plots using regular plus signs. In **B** and **C**, the statistical significance of Han1k_KP compared to Han1k_HP is indicated by italic plus signs. **D** Significantly differential species between Han1k_KP and Han4k_w. **E** Heat maps of significantly differential species increased in Han4k_6m and Tibetan4k_NP compared to Han1k_KP, while decreased in Han4k_d3m compared to Han4k_6m. **F** Heat maps of significantly differential species decreased in Han4k_6m and Tibetan4k_NP compared to Han1k_KP, while increased in Han4k_d3m compared to Han4k_6m. Heat maps are scaled by row. The statistical significance of species between two groups is marked by plus signs. The *q* value < 0.05 denoted as “ + ”; the *q* value < 0.01 denoted as “ +  + ”; the *q* value < 0.001 denoted as “ +  +  + ”

To further investigate the microbial signatures, differential analysis of species was performed across groups (Fig. 2A, Supplementary Table 4). Only 39 species exhibited differential abundance between Han1k_KP and Han1k_HP, while consistent variations were observed in 186 species between Tibetan4k_NP and both Han1k_KP and Han1k_HP. Among these, 111 species were enriched in Tibetan4k_NP, whereas 75 species showed enrichment in both Han1k_KP and Han1k_HP. The top 20 significantly enriched species in Tibetan4k_NP are presented (Fig. 2B). In addition to *Prevotella* spp., *Alistipes* sp. *CAG:435*, and *Alistipes* sp. *CAG:514* were significantly enriched in Tibetan4k_NP. Strain genome assembly results revealed that both *Alistipes* sp. *CAG:435* an*d Alistipes* sp. *CAG:514* exhibited gene functions associated with thermogenesis for environmental adaptation (Supplementary Fig. 1C and D). Moreover, the butyrate-producer *Butyrivibr*io *crossotus*, which is inversely associated with obesity [17], displayed a substantially higher abundance (28-fold) in Tibetan4k_NP compared to Han1K_KP group. The top 20 significantly enriched species in both Han1k_KP and Han1k_HP showed that opportunistic pathogens, such as *Kluyvera ascorbate*, *Klebsiella pneumoniae*, *Salmonella enterica*, *Escherichia coli*, and *Shigella*, were significantly increased (Fig. 2C).

To identify specific species associated with the acute response stage, a comparison was conducted between Han4k_1w and Han1k_KP, resulting in the identification of only 14 differential species (Fig. 2D). Notably, *Escherichia coli*, *Klebsiella pneumoniae*, *Kluyvera ascorbate*, and *Shigella* were significantly decreased in Han4k_1w. Conversely, the abundances of *Ruthenibacterium lactatiformans* and *Clostridium leptum* were higher in Han4k_1w. Furthermore, *Hungatella hathewayi*, which has been positively correlated with circulating taurine concentration in humans [18], showed significant enrichment in Han4k_1w.

To further identify the species associated with acclimatization and deacclimatization, differential analyses were conducted between Han1k_KP and Han4k_6m, as well as between Han4k_6m and Han4k_d3m. A total of 176 differential species were identified during the acclimatization stage, out of which 81 showed significant increase in abundance in Han4k_6m (Fig. 2A “Han4k_6m vs Han1k_KP”). During the deacclimatization stage, a total of 228 differential species were identified, among which 120 species were significantly enriched in Han4k_d3m (Fig. 2A “Han4k_d3m vs Han4k_6m”). Further attention was given to those species that consistently varied between Han1k_KP or Han4k_d3m and Han4k_6m or Tibetan4k_NP.

In total, there were 51 species that showed significant increase in both Han4k_6m and Tibetan4k_NP compared to that of Han1k_KP, while decreased in Han4k_d3m compared to Han4k_6m (Fig. 2E). *Prevotella copri*, the most prevalent member within *Prevotella* genus, exhibited a significant increase in both Han4k_6m and Tibetan4k_NP (Supplementary Fig. 1E). Some species known for their ameliorative effects on diseases, such as *Bacteroides xylanisolvens* alleviating smoking-related non-alcoholic steatohepatitis [19], *Bacteroides faecis* improving the epithelial barrier integrity [20], and *Parabacteroides distasonis* alleviating inflammatory arthritis, obesity, and metabolic dysfunctions [21, 22], showed significant increase during acclimatization stage. Furthermore, immune-related species, such as *Bacteroides intestinalis* enhancing host immune response [23], *Bacteroides ovatus* and *Prevotella buccalis* inducing IgA production [24, 25], and *Odoribacter splanchnicus* promoting intestinal Th17 cell development [26], were significantly increased. Additionally, the enrichment of crucial players known for their abilities to degrade complex molecules like cellulose, hemicellulose, and xylan, such as *Bacteroides cellulosilyticus*, *Prevotella bryantii*, and *Paraprevotella xylaniphila*, were also observed.

In contrast, 57 species showed significant decreases in both Han4k_6m and Tibetan4k_NP compared to Han1k_KP, but increased in Han4k_d3m (Fig. 2F). Notably, during the deacclimatization stage, *Escherichia coli*, *Klebsiella pneumoniae*, *Kluyvera ascorbate*, and *Shigella* showed significant increases. *Faecalibacterium prausnitzii*, one of the top 10 most abundant species (Supplementary Fig. 1E) and a crucial butyrate-producing bacterium in human colon, significantly increased in Han4k_d3m. Short-chain fatty acid producers, such as *Subdoligranulum variabile*, *Coprococcus comes*, *Holdemania filiformis*, *Clostridium leptum*, *Anaerotruncus colihominis*, *Butyricicoccus pullicaecorum*, and *Marvinbryantia formatexigens*, were also significantly elevated. Species belonging to *Bifidobacterium* like *Bifidobacterium adolescentis*, *Bifidobacterium pseudocatenulatum*, *Bifidobacterium longum*, and lactic acid producers like *Lactococcus lactis*, *Ruthenibacterium lactatiformans*, and *Enterococcus faecium* were all significantly increased. *Blautia*, a new functional genus, exhibited an increase in species abundance, such as *Blautia wexlerae*, *Blautia obeum*, and *Blautia producta*. Additionally, *Dorea longicatena* and *Tyzzerella nexilis*, as potential biomarkers of obesity and type 2 diabetes [27, 28], as well as *Clostridioides difficile* and *Vibrio parahaemolyticus* known to cause diarrhea, were all found to be significantly increased.

### Identification of Tibetan and Han populations based on gut microbiota

We subsequently developed an eXtreme Gradient Boosting (XGBoost) classifier using a dataset consisting of 138 Tibetan and 472 Han individuals from all cohorts. To determine the optimal feature combination for classifying Tibetan and Han populations across all species, we conducted ten iterations of tenfold cross-validation on the training data. Employing a selection of 24 species, our model achieved an AUC of 0.96 (*p* < 1e-05) in the test data, effectively discerning between Tibetan and Han individuals (Supplementary Fig. 2A). Furthermore, a new model established to classify 232 Hans residing on the plain (Han1k_KP, Han1k_HP) and 63 Hans residing on the plateau (Han4k_6m). It attained a test data AUC value of 0.96 (*p* < 1e-05) based on 17 species features (Supplementary Fig. 2B).

### Characteristics of plasma metabolome and clinical indices

**Fig. 3:**
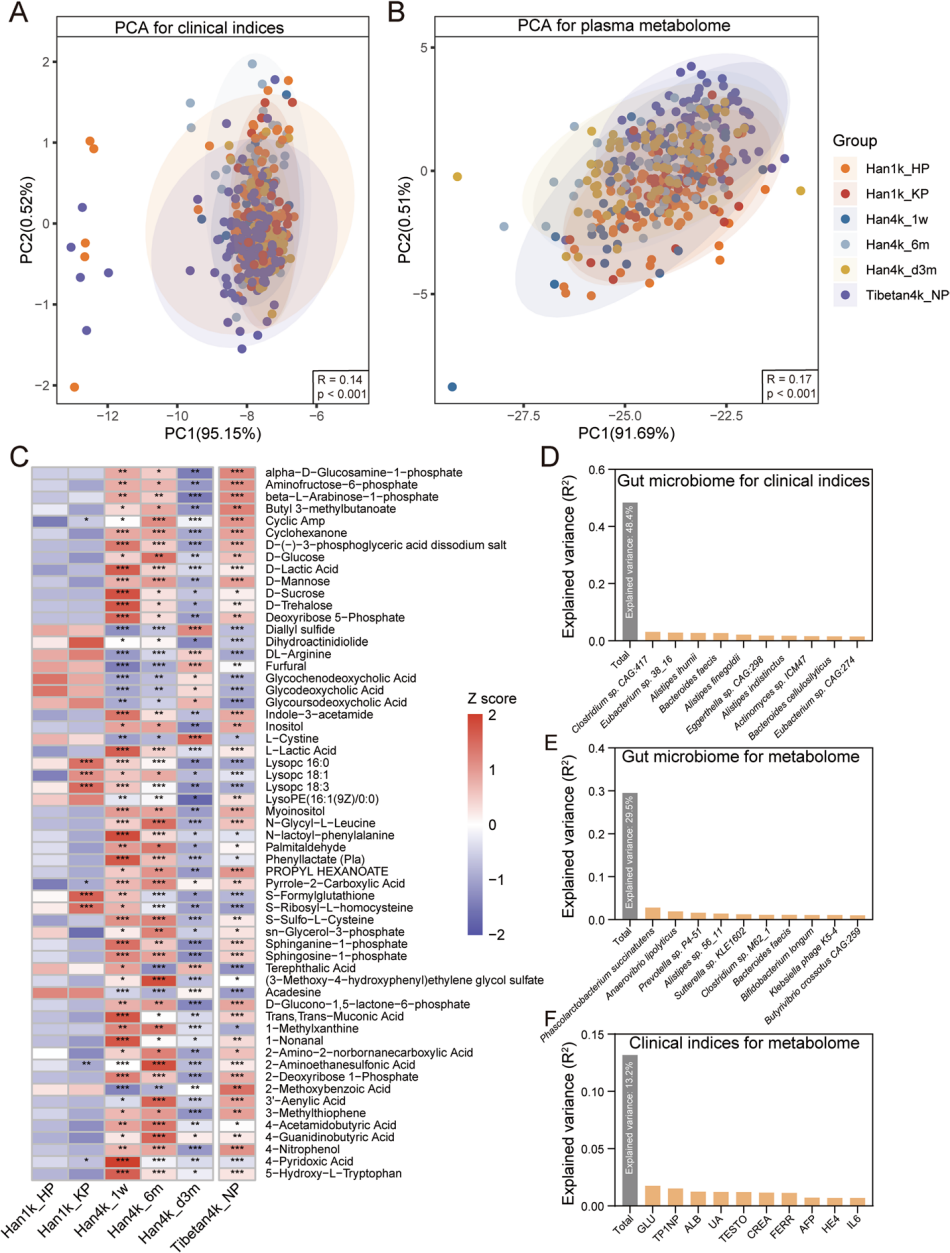
Plasma clinical indices and metabolome analysis. **A** PCA for plasma clinical indices. **B** PCA for plasma metabolome. Anosim test based on Bray–Curtis distance was conducted to measure the dissimilarity across groups. **C** Significantly differential plasma metabolites in both time-altitude cohort and Tibetan4k_NP. The plus signs in column Han1k_KP, Han4k_1w, Han4k_6m, Han4k_d3m, and Tibetan4k_NP represent significance of the comparison between Han1k_KP and Han1k_HP, between Han4k_1w and Han1k_KP, between Han4k_6m and Han1k_KP, between Han4k_d3m and Han4k_6m, and between Tibetan4k_NP and Han1k_KP, respectively. The *q* values < 0.05 denoted as “ + ”; the *q* values < 0.01 denoted as “ +  + ”; the *q* values < 0.001 denoted as “ +  +  + .” **D** The top 10 representative species with the highest explanation of the plasma clinical indices. **E** The top 10 representative species with the highest explanation of the plasma metabolome. **F** The top 10 representative plasma clinical indices with the highest explanation of the plasma metabolome. The total variance in clinical indices or metabolome explained by gut microbiome or clinical indices is displayed by the gray column

To further quantify clinical indices and plasma metabolites, we conducted clinical testing and widely targeted metabolome analyses on 418 and 403 plasma samples, respectively. The demographic and clinical characteristics of each group are presented in Table 2. Based on 65 clinical indices, principal component analysis (PCA) revealed significant differences across groups (Fig. 3A, Anosim test, *R* = 0.14, *p* < 0.001). During the acute response stage, Han4k_1w showed significantly elevated levels of ProBNP, FPSA, TNT-HS, Insulin, NH3, APOA, TRIGL, CHO, HDL, T4, and A-HCV, whereas CRPHS, CK, ALT, CORT, LIPC, AST, and MYO levels were decreased. During the acclimatization stage, Han4k_6m had significantly higher levels of CA153, LDIP, TESTO, E2, AST, CPEPTID, APOB, FT4, T4, HDL, TP, and ALB compared to Han1k_KP and Han4k_d3m, whereas GLU, PCT, FERR, PRL, PROG, ProBNP, and CRPHS were decreased. Tibetan4k_NP specifically had higher GGT, HE4, ProGRP, TP1NP, and TSH than Han1k_KP, while no significant differences were observed between other groups. Notably, IL6, BILT, AMY-L, E2, TESTO, FT4, and FT3 were significantly higher in Han4k_1w, Han4k_6m, and Tibetan4k_NP than in Han1k_KP, whereas UA, GLU, and PHO were lower.

A total of 629 plasma metabolites were quantified, and PCA showed significant separation of the plasma metabolome among groups (Fig. 3B, Anosim test, *R* = 0.17, *p* < 0.001). The 59 metabolites with significant differences in both time-altitude cohort and Tibetan4k_NP are displayed (Fig. 3C). Among these, 41 metabolites were significantly elevated in Han4k_1w, Han4k_6m, and Tibetan4k_NP compared to Han1k_KP but decreased in Han4k_d3m compared to Han4k_6m. In contrast, 8 metabolites exhibited opposite trends. Notably, lactic acid, D-mannose and D-sucrose, and D-trehalose levels increased significantly after ascending to high altitude. Additionally, sphingosine-1-phosphate and sphinganine-1-phosphate levels were upregulated by high altitude exposure. Sphingosine-1-phosphate has been reported to confer adaptive and protective advantages against hypobaric hypoxia during acute or sub-chronic phases [29]. Plasma taurine (2-aminoethanesulfonic acid) levels also showed a significant increase at high altitude, which also has been shown to have protective effects against hypoxia [30, 31]. Inositol elevated in the frontal cortex of hypoxia-treated rats [32] was increased at high altitude. Conversely, metabolites involved in bile acid metabolism including glycochenodeoxycholic acid, glycodeoxycholic acid, and glycoursodeoxycholic acid exhibited significant decreases in Han4k_1w, Han4k_6m, and Tibetan4k_NP.

### Altered plasma clinical indices and metabolome are associated with gut microbiota at high altitude

An effect size analysis was conducted to evaluate the proportion of variance in clinical indices or metabolome explained by gut microbiome or clinical indices. The total effect size of gut microbiota on plasma clinical indices accounted for 48.4% of the variance and the top 10 species with the highest explanation were displayed (Fig. 3D). The total effect size of gut microbiota on plasma metabolome accounted for 29.5% of the variance (Fig. 3E). However, compared to that of the gut microbiota, the effect size of plasma clinical indices on metabolome was smaller, and only 13.2% metabolome variance could be explained by clinical indices (Fig. 3F). The top 10 clinical indices with the highest explanation of the metabolome showed that GLU, TP1NP, ALB, UA, and IL6 exerted significant effects on the variance of plasma metabolome. These results suggest that at high altitude, gut microbiota plays an important role in determining both plasma clinical indices and metabolic landscape.

Correlation analysis was performed in both plain populations (Han1k_HP, Han1k_KP) and plateau populations (Han4k_1w, Han4k_6m, Han4k_d3m, and Tibetan4k_NP). In the plain populations, we identified 82 correlations between 16 clinical indices and 48 species (Supplementary Fig. 3A). IL6 exhibited the highest degree of connectivity and was negatively correlated with opportunistic pathogens such as *Shigella* spp., *Salmonella enterica*, and *Escherichia coli*, while positively correlated with *Blautia* spp. In contrast, in plateau populations, a total of 134 correlations were found between 18 clinical indices and 100 species (Supplementary Fig. 3B). TP1NP and UA were the two nodes with the highest degree of connectivity that correlated with 50 and 25 species respectively. Interestingly, no correlation was observed between TP1NP levels and gut microbiota in plain populations. Notably, at high altitude however, TP1NP that is considered a specific marker for bone formation showed positive correlation with *Prevotella* sp. *CAG:474* and *Alistipes* sp. *CAG:51*. Additionally, it was also positively correlated with microbial ascorbate (M00114), phosphatidylethanolamine (M00093), and tetrahydrofolate (M00841) biosynthesis (Supplementary Fig. 4). Furthermore, plasma GLU levels were positively associated with F-type ATPase (M00158), which generates adenosine triphosphate as the universal major energy source, indicating possible regulation by gut microbiota at high altitude.

In plain populations, 538 correlations between gut microbiota and plasma metabolome showed 118 metabolites that were significantly correlated with 184 species (Supplementary Fig. 3C). Methyl 3-hydroxyphenylacetate and acetaminophen glucuronide were the two nodes with the largest degrees. In plateau populations, 2446 correlations showed that 79 metabolites were significantly correlated with 204 species (Supplementary Fig. 3D). Notably, 760 correlations between 137 specific nodes were exclusively identified in plateau populations, and most species belonged to *Prevotella*. Guanosine 5′-diphosphate (GDP), terephthalic acid, and thymidine were the three largest degree nodes, and their associations with gut microbiota were only identified in plateau populations. Increased correlations suggest that the associations between plasma metabolites and gut microbiota are significantly enhanced at high altitude. Correlations between creatine and gut microbiota were specifically identified in plateau populations and were positively correlated with *Blautia hansenii* and *Clostridioides difficile*. Creatine can enhance corticomotor excitability and cognitive performance during oxygen deprivation [33]. We also found that creatine levels were significantly higher in Han4k_1w than in Han1k_KP through plasma metabolome analysis (Supplementary Fig. 6F). Caffeic acid, which is a novel hydroxylase-2 inhibitor that upregulates hypoxia-inducible factors and provides protection against hypoxia [34], was positively correlated with *Firmicutes bacterium CAG:110* and *Oscillibacter* sp. *CAG:241_62_21* in both plain and plateau populations. The caffeic acid levels in Tibetan4k_NP were also significantly higher than Han1k_KP (Supplementary Fig. 6G). Hyodeoxycholic acid and glycoursodeoxycholic acid both were positively correlated with *Clostridium* sp. *AT4* and *Ruminococcus gnavus*, while negatively correlated with *Alistipes* spp. and *Prevotella* spp. The plasma levels of sphingosine-1-phosphate were negatively correlated with *Streptococcus thermophilus* and *Dorea longicatena*, while displaying the most associations with microbial module pathways (Supplementary Fig. 5).

### Altered microbial functions in acclimatization and deacclimatization stages

To further investigate the changes in microbial functions during acclimatization and deacclimatization stages, differential function analysis was conducted between Han1k_KP and Tibetan4k_NP, Han1k_KP and Han4k_6m, and between Han4k_6m and Han4k_d3m. A total of 524 Kyoto Encyclopedia of Genes and Genomes (KEGG) modules were annotated, among which 418 modules were identified as differentially abundant between Tibetan4k_NP and Han1k_KP (Supplementary Table 5). Notably, we observed a significant enrichment of the cAMP signaling module (M00695), which plays an important role in protecting against hypoxia-induced impairment [35], in Tibetan4k_NP. Additionally, the incomplete reductive citrate cycle of methanogenic archaea (M00620) was significantly increased in Tibetan4k_NP, and it was further validated by the enrichment of Methanobacteriaceae (Supplementary Fig. 1F). Previous studies have showed that methanogens can convert pyruvate to the necessary biosynthetic intermediates in response to anaerobic or microaerophilic growth conditions [36]. We speculate that the enrichment of methanogens in Tibetan populations may help their adaptation to high altitude.

**Fig. 4:**
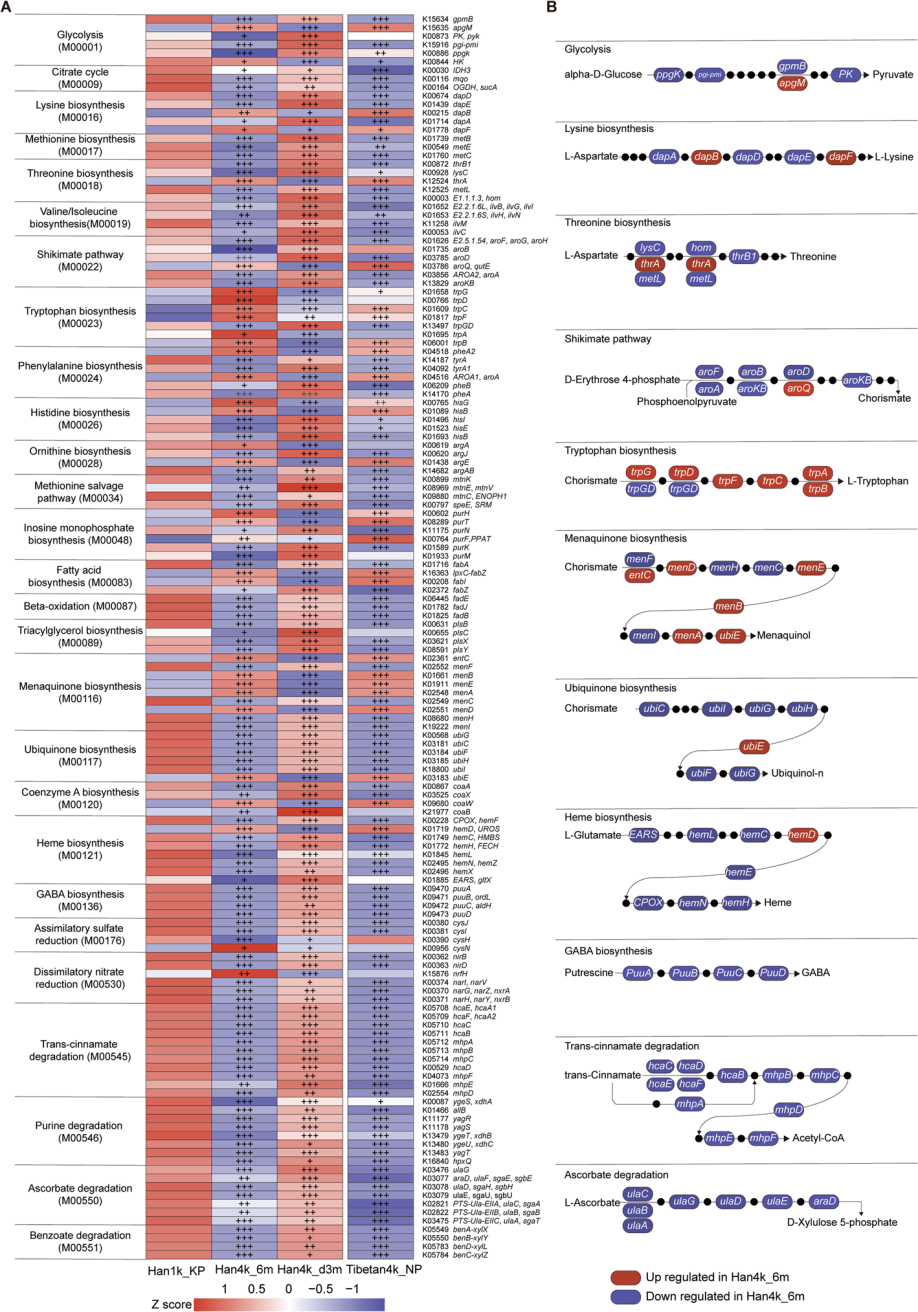
Altitude-associated changes in microbial genes summarized in KO genes and KEGG pathway modules. **A** Heat maps of 146 significantly differential KO genes between Han4k_6m and Han1k_KP and Han4k_d3m and Han4k_6m. KO genes annotated in the same modules are clustered and marked. Heat maps are scaled by row. **B** Representative KO genes appearing in **A** are shown in pathway modules modified from KEGG pathway maps “Glycolysis,” “Lysine biosynthesis,” “Threonine biosynthesis,” “Shikimate pathway,” “Tryptophan biosynthesis,” “Menaquinone biosynthesis,” “Ubiquinone biosynthesis,” Heme biosynthesis,” “GABA biosynthesis,” “Trans-cinnamate degradation,” and “Ascorbate degradation.” Each box in a pathway represents a KO gene and is marked in red for elevation in Han4k_6m or in blue for depletion in Han1k_KP and Han4k_d3m. Black dots in each pathway represent intermediate metabolites. The statistical significance between two groups is marked by plus signs. The *q* values < 0.05 denoted as “ + ”; the *q* values < 0.01 denoted as “ +  + ”; the *q* values < 0.001 denoted as “ +  +  + ”

Differential analysis identified 353 differential KEGG modules between Han1k_KP and Han4k_6m, as well as 393 differential KEGG modules between Han4k_6m and Han4k_d3m (Supplementary Table 5). To further investigate the metabolism-related microbial functions, we selected and presented 146 significantly differential KEGG orthology genes (KO genes) between Han4k_6m and Han1k_KP, as well as between Han4k_d3m and Han4k_6m (Fig. 4A). Among these genes, a total of 145 KO genes showed reverse changes in abundance in Han4k_6m and Han4k_d3m, indicating their possible regulation by altitude. Microbial pathway modules enriched with differentially abundant KO genes were manually constructed (Fig. 4B). Notably, the KO genes involved in glycolysis (M00001) showed a significant decrease in Han4k_6m. Specifically, the rate-limiting enzyme like pyruvate kinase (*PK*) was significantly decreased in Han4k_6m but increased in Han4k_d3m. Furthermore, two rate-limiting enzymes involved in the citrate cycle (M00009), namely 2-oxoglutarate dehydrogenase (*OGDH*) and isocitrate dehydrogenase (*IDH3*), were decreased in both Han4k_6m and Tibetan4k_NP. Additionally, downregulation of genes involved in ubiquinone biosynthesis, a crucial component of bacterial electron transfer chains, and heme biosynthesis, a precursor to hemoglobin necessary for oxygen transport in the bloodstream, were observed in Han4k_6m and Tibetan4k_NP. These results suggest that, similar to that observed in Tibetans, the gut microbial aerobic respiration was also inhibited during acclimatization to high altitude in Han populations.

During the acclimatization stage, Han4k_6m showed significant decreases in KO genes involved in methionine biosynthesis (M00017), methionine salvage pathway (M00034), threonine biosynthesis (M00018), and valine/isoleucine biosynthesis (M00019), indicating a reduction in the gut microbial capacity to synthesize these amino acids. Moreover, microbial genes involved in the shikimate pathway (M00022) for folate and aromatic amino acid synthesis were notably decreased, as were those associated with beta-oxidation (M00087) and triacylglycerol biosynthesis (M00089). Four genes responsible for γ-aminobutyric acid (GABA) production were also downregulated in Han4k_6m and Tibetan4k_NP. Bacteria have been shown to produce and/or consume GABA, which may affect host physiology [37]. Interestingly, plasma metabolome analysis revealed elevated levels of GABA in Han1k_HP and Han1k_KP but reduced levels in Tibetan4k_NP (Supplementary Fig. 6D). Ascorbate degradation was significantly reduced in Han4k_6m, which is vital for regulating transcription factor hypoxia-inducible factor activity [38]. Specifically, tryptophan biosynthesis, the precursor of the neurotransmitter serotonin, was significantly enriched in Han4k_6m, while the levels of tryptamine, another bacterial-derived tryptophan catabolite that stimulates the release of serotonin by enterochromaffin cells [39], showed a significant increase in Han4k_6m (Supplementary Fig. 6E).

### Gut microbiota regulates host purine metabolism at high altitude

**Fig. 5:**
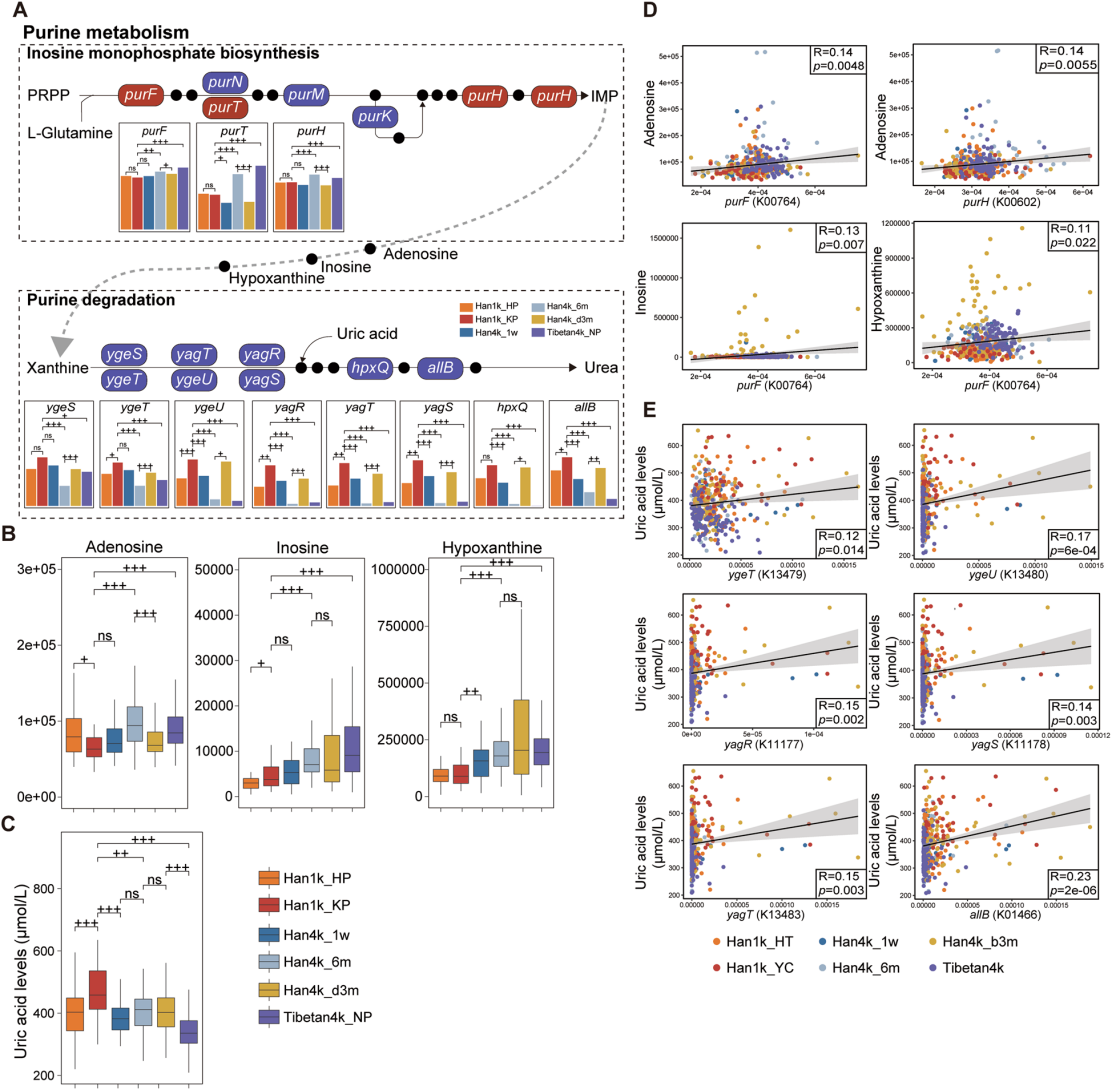
Gut microbiota fluctuated with altitude is associated with purine metabolism. **A** Similar to Fig. 4B, significantly differential KO genes involved in purine metabolism are shown in pathway modules modified from KEGG pathway maps “Inosine monophosphate biosynthesis” and “Purine degradation.” Bar plots show relative gene abundances in different groups. **B** Plasma metabolite levels analyzed by widely targeted mass spectrometry in different groups. **C** Plasma UA levels detected by clinical test. The statistical significance between two groups is marked by plus signs. The *q* values < 0.05 denoted as “ + ”; the *q* values < 0.01 denoted as “ +  + ”; the *q* values < 0.001 denoted as “ +  +  + .” **D** Pearson correlation analysis between relative abundance of genes and purine metabolites levels. **E** Pearson correlation analysis between relative abundance of genes and plasma UA levels

We observed a significant upregulation of amidophosphoribosyl transferase (*purF*), the rate-limiting enzyme in inosine monophosphate (IMP) biosynthesis, and IMP cyclohydrolase (*purH*) in Han4k_6m and Tibetan4k_NP, while observing downregulation in Han1k_KP and Han4k_d3m (Fig. 5A). Additionally, we found a notable decrease in eight genes involved in purine degradation in Han4k_6m and Tibetan4k_NP, while they increased in Han1k_KP and Han4k_d3m. These alterations suggest an elevation of IMP biosynthesis but inhibition of its degradation at high altitude. We speculate that the aberrant microbial purine metabolism at high altitude may be associated with host blood UA levels.

The metabolome analysis revealed a significant increase in the levels of adenosine, inosine, and hypoxanthine in Han4k_6m and Tibetan4k_NP (Fig. 5B). Clinical testing results demonstrated that plasma UA levels were significantly higher in Han1k_KP compared to other groups (Fig. 5C). Conversely, Tibetan4k_NP exhibited significantly lower UA levels than other groups. Furthermore, the UA levels of Han4k_1w, Han4k_6m, and Han4k_d3m were significantly lower than those observed in Han1k_KP. These results indicate a significant reduction in blood UA levels of both Tibetan and Han populations after ascending to high altitude in our study cohorts.

Pearson’s correlation analysis revealed positive associations between the abundance of *purF* and plasma adenosine, inosine, and hypoxanthine levels (Fig. 5D), indicating the involvement of KO genes in IMP biosynthesis. Furthermore, the KO genes involved in purine degradation, namely *ygeT*, *ygeU*, *yagR*, *yagS*, *yagT*, and *allB*, were found to be positively correlated with plasma UA levels (Fig. 5E). To further investigate the key species associated with plasma UA levels, a correlation analysis was conducted between species and KO genes (Supplementary Fig. 6H, rho > 0.6). The results demonstrated strong positive correlations between seven species including *Shigella dysenteriae*, *Shigella sonnei*, *Shigella flexneri*, *Klebsiella pneumoniae*, *Kluyvera ascorbate*, *Salmonella enterica*, and *Escherichia coli* with xanthine dehydrogenase (*yagR*, *yagS*, *ygeU*, *yagT*). Notably, *Escherichia coli* in the intestine secretes xanthine dehydrogenase which is an important rate-limiting enzyme responsible for oxidative metabolism of purines [40]. These seven species also exhibited positive correlations with allantoinases (*allB*, *hpxB*, EC:3.5.2.5, K01466, K16842). *Klebsiella pneumoniae* was positively correlated with 2-oxo-4-hydroxy-4-carboxy-5-ureidoimidazoline decarboxylase (*hpxQ*). Positive correlations between *Escherichia coli*, *Klebsiella pneumoniae*, and plasma UA levels were also observed (Supplementary Fig. 6AB).

**Fig. 6:**
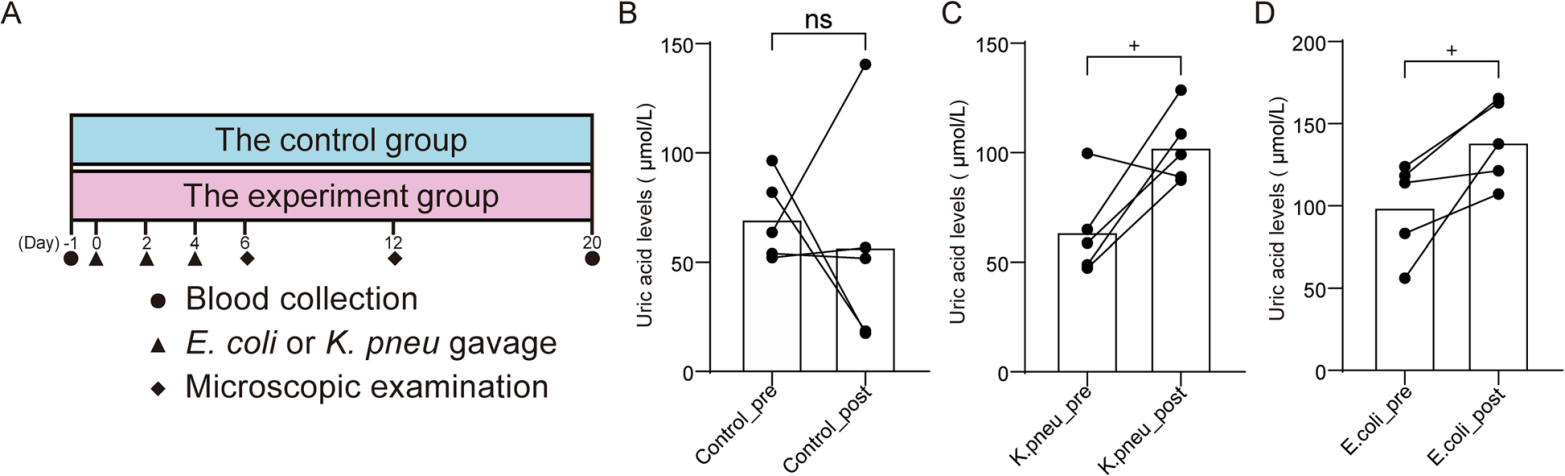
Host plasma UA levels regulated by *Escherichia coli* (*E. coli*) and *Klebsiella pneumoniae* (*K. pneu*). **A** Animal experimental design for germ-free mice with administration of *E. coli* or *K. pneu*. **B** Paired *t* test for plasma UA levels of control group between before and after gavage with saline. **C** Paired *t* test for plasma UA levels of experiment group between before and after gavage with *K. pneu*. **D** Paired *t* test for plasma UA levels of experiment group between before and after gavage with *E. coli*. The *p* values < 0.05 denoted as “ + ”; the *p* values > 0.05 denoted as “ns”

To establish the causal relationship between *Escherichia coli* or *Klebsiella pneumoniae* and plasma UA levels, germ-free mice administered via gavage with these two species and plasma UA levels were analyzed using a mouse UA ELISA kit. The experimental design is illustrated in Fig. 6A. In the control group, no significant difference was observed in plasma UA levels before and after administration (Fig. 6B). As anticipated, gavage with *Klebsiella pneumoniae* and *Escherichia coli* resulted in a substantial increase in plasma UA levels (Fig. 6CD). Hence, the elevated abundance of *Escherichia coli* and *Klebsiella pneumoniae* in the gut contributes to elevated host plasma UA levels.

## Discussion

Our study reveals the dynamics of human gut microbiota during acute response, acclimatization, and deacclimatization to high altitude, providing new evidence for the resilience of microbial communities under altitude-induced perturbations. With prolonged residence time on the plateau, the gut microbial structure of Han individuals gradually approached that of Tibetans, suggesting that there is a representative enterotype in high-altitude populations. Given the adaptive enterotype of Tibetans to high altitude, “assimilation” of gut microbiota towards Tibetan may aid Han individuals in acclimatizing to high altitude. Similar to Tibetan populations, the abundance of *Prevotella* significantly elevated during the acclimatization stage in Han populations, suggesting its potential as a representative genus in high-altitude populations. A hypoxic environment can lead to an increase in anaerobes in the gut [41]. *Prevotella* spp., as obligate anaerobes, have a competitive advantage over aerobes and thus being more likely to be abundant in human gut at high altitude. Additionally, in rural populations who follow a more pre-industrial and traditional lifestyle, *Prevotella* tends to dominate the gut microbiota [42]. We speculate that the abundant *Prevotella* in high-altitude populations is partly associated with low industrialization levels on the Tibetan Plateau. Our study suggests that after acclimatizing to high altitude, the gut microbiota of the Hans shares similar characteristics with those of Tibetans. However, further mechanistic studies are required to determine whether this feature affects host adaptation at high altitude.

As previously reported, host genetic effects on gut microbiota are universal [43]. Given the prolonged high-altitude habitation of Tibetans, natural selection has led to an enrichment of unique genotypes. Notably, *EPAS1* exhibits a highly distinctive haplotype structure exclusively found in Tibetans and Denisovans, with a very low frequency among Han Chinese [44]. Based on large-scale Tibetan whole-genome sequencing data, a high-confidence list of 4320 variants and 192 genes that have undergone selection in Tibetans has been characterized [45]. The variation in gut microbiota is influenced by both environmental and genetic factors. However, multiple studies consistently indicate that environmental effects exert a greater impact than additive genetic effects on gut microbiota [43, 46]. In our study, Han and Tibetan populations cohabitated under identical altitudes, diets, and lifestyles conditions. It is plausible that genetic effects potentially contributed to shaping the distinct characteristics of gut microbiota between these two populations despite their prolonged cohabitation. Therefore, we postulated that despite prolonged cohabitation, the gut microbiota of the two populations would exhibit similarities while still retaining discernible distinctions.

Many SCFA producers were elevated in Tibetan and Han populations after acclimatization to high altitude. Among them, *Prevotella* spp. are proficient in producing SCFA propionate from arabinoxylans and fructo-oligosaccharides [16]. The increased production of SCFA could provide more efficient energy intake for high-altitude populations compared to plain populations. *Alistipes* sp. *CAG:435* and *Alistipes* sp. *CAG:514*, two other highly enriched SCFA producers found in Tibetans with genomic functions favoring thermogenesis for environmental adaptation, may be beneficial for adaptation to the low temperature of Tibetan Plateau as well. Cold-exposed microbiota increased concentrations of SCFA and serum ghrelin levels, thus increasing energy intake and thermogenesis in host adaptation to cold [47]. Therefore, the increased SCFA producers resulting from chronic exposure to high altitude may contribute to host acclimatization to such conditions.

Firmicutes increased significantly in the Han population, while Bacteroidetes increased significantly in the Tibetan population. Previous study has shown that Firmicutes is positively associated with obesity while Bacteroidetes is negatively associated with it [48]. In Tibetans, we also found an increase of microbes known to ameliorate type 2 diabetes or obesity, such as *Butyrivibrio crossotus*, *Prevotella copri*, and *Parabacteroides distasonis*. Moreover, a higher ratio of *Prevotella* to *Bacteroides* was observed in subjects who showed improved GLU metabolism [49]. Given that Tibetans have lower incidence rates of diabetes and obesity [50, 51], we speculate that this may be related to their unique microbial composition. Moreover, the decreased plasma GLU levels of the Han after ascending to the high altitude might be partly attributed to their gut microbiota approaching that of the Tibetan (Supplementary Fig. 6C). Consequently, future studies focusing on associations between gut microbiota and blood GLU levels among Tibetans may identify more potential probiotics for treating type 2 diabetes.

We identified 51 species that were elevated and 57 species that decreased at high altitude. These species were validated through comparisons across different cohorts and were found to be independent of ethnicity, suggesting their critical responses to altitude. Most of the elevated species belonged to *prevotella* and *Bacteroides*, indicating that they are more adaptive to high altitude. Conversely, we found a decrease in some opportunistic pathogens, such as *Escherichia coli, Klebsiella pneumoniae*, *Shigella*, *Salmonella enterica*, and *Clostridioides difficile*, in high altitude populations. This suggests that populations residing at higher altitudes may face a lower risk of infection from these pathogens compared to those living in lowland areas.

We identified 41 significantly enriched metabolites in Han population after ascending to high altitude, which may contribute to acclimatization. Notably, sphingosine-1-phosphate and taurine, both of which have protective effects against hypobaric hypoxia exposure, were significantly increased in Tibetan and Han populations after ascending to the plateau. Moreover, sphingosine-1-phosphate was positively correlated with 58 microbial module pathways, such as the PTS system, valine/isoleucine biosynthesis, fatty acid biosynthesis, and leucine biosynthesis; thus, the associations between sphingosine-1-phosphate levels and gut microbiota at high altitude warrant further exploration.

The reduced microbial biosynthesis of acetyl-CoA, pyruvate, citrate, and heme in high altitude populations suggests that aerobic respiration of gut microbiota was inhibited. Oxygenation by the bloodstream is closely related to the gut O_2_ environment [52], thus may result in a decrease in aerobic bacteria and an increase in anaerobic bacteria [53]. Additionally, we observed a decrease in GABA biosynthesis-related genes in Han4k_6m and Tibetan4k_NP. Downregulated GABA levels in plasma were also observed in the Tibetans. GABA is the primary inhibitory neurotransmitter in the brain and its levels are associated with anxiety and depression [54]. Although the abundance of fecal GABA producers such as *Bacteroides* was negatively correlated with depression signatures [55], it remains unclear whether gut-derived GABA can cross the blood–brain barrier and improve mood [56]. Exhilaratingly, two GABA-producing *Lactobacillus* strains have been shown to reduce depression-like behavior in mice [57]. At high altitude, one’s mood tends to change and the exacerbation of depression was concluded [58], and that may be related to a decrease in gut microbes harboring genes responsible for GABA biosynthesis.

Remarkably, IMP biosynthesis in purine metabolism was enhanced at high altitude, while purine degradation was inhibited. Plasma inosine, a purine nucleoside known for its neuroprotective effects against hypoxia [59], increased at high altitude. Hypoxia and reoxygenation have also been reported to elevate inosine levels [60]. Inosine exerts significant cytoprotective action under hypoxic conditions and surprisingly serves as a more efficient energy source than equivalent amounts of GLU [61]. Therefore, the elevated inosine levels observed at high altitude are crucial for protecting against hypoxia and providing energy, making it a potential therapeutic agent for acclimatization to high altitude. IMP is one of the precursors involved in inosine synthesis. Further verification is required to determine whether increased IMP synthesis by gut microbiota promotes plasma inosine production.

Our study identified several species strongly positively associated with microbial genes involved in purine degradation, such as *Shigella*, *Klebsiella pneumoniae*, *Kluyvera ascorbate*, *Salmonella enterica*, and *Escherichia coli*, which were decreased at high altitude. The study has provided evidence that *Escherichia coli* is capable of secreting xanthine dehydrogenase(XOD) [40], a crucial rate-limiting enzyme responsible for the oxidative metabolism of purine. Although these species are opportunistic pathogens, our study indicates that they may play crucial roles in purine degradation at high altitude. The prevalence of hyperuricemia is higher in Tibetan compared to lowlanders [62, 63]. However, its occurrence is influenced by various factors including dietary and cardiovascular risk factors rather than solely being determined by altitude alone [64]. Our research further demonstrates that plasma UA levels are closely associated with alterations in gut microbial composition at high altitude.

In previous study, stool samples were collected at four time points throughout the year, with 3-month intervals between each collection, and found a distinct seasonal cycle in the gut microbiota of the Hadza [65]. Moreover, it was discovered that significant alterations in gut microbiota can occur within a 3-month period following seasonal changes. This study suggests that changes in the external environment are sufficient to alter the gut microbiota within 3 months. Additionally, it is commonly observed that after residing at high altitudes for approximately 3 months, symptoms associated with altitude sickness subside and individuals reach a state where their respiration and pulse rates resemble those experienced at lower altitudes. Consequently, sampling intervals of 1 week, 3 months, and 6 months provide sufficient data for investigating short-term responses to high altitude. However, a comprehensive investigation into the long-term adaptation of gut microbiota to chronic high altitude exposure may necessitate a duration of 1 year or more.

This study has several limitations. Firstly, our study exclusively focused on investigating the impact of high altitude at 4300 m, without considering multiple altitude gradients. Given the variations in oxygen concentration, ultraviolet radiation, and air temperature across different altitudes, it is plausible that the influence on gut microbiota may exhibit varying magnitudes. Consequently, our study does not provide a comprehensive representation of the changes in gut microbiota across all high-altitude gradients. Secondly, our study primarily focused on the short-term response of gut microbiota to high altitude, excluding individuals who had resided at high altitudes for more than a year or had prolonged exposure to such conditions. It is important to note that chronic mountain sickness may develop with long-term exposure to high altitude, thus necessitating further investigation into the alterations in gut microbiota under prolonged exposure, which were beyond the scope of this study. Thirdly, our study revealed a tendency for the gut microbiota of Han to converge towards that of Tibetan after residing at high altitudes for 6 months. However, it is important to note that while Tibetan serves as an exemplary model of successful adaptation to high altitude, the convergence in gut microbiota composition with Tibetan does not necessarily imply facilitation of high-altitude adaptation in Han. Further investigations are warranted to validate these findings. In our future studies, greater emphasis will be placed on elucidating the mechanisms through which gut microbiota contributes to host adaptation to high altitude.

## Conclusions

In conclusion, our study unveiled dynamic fluctuations in gut microbiota of the Han as they migrated from low altitude to high altitude and back again. After residing at high altitude for 6 months, the composition of their gut microbiota gradually converged towards that of the Tibetan. Altitude exerted a significant impact on both plasma metabolome and clinical indices, with a substantial portion of their variance being explained by alterations in gut microbiota. Notably, key players involved in purine degradation in gut microbiota, such as *Escherichia coli* and *Klebsiella pneumoniae*, played crucial roles in regulating host plasma UA levels at high altitude.

### Supplementary Information

**Supplementary Material 1.**

**Supplementary Material 2.**

**Supplementary Material 3.**

**Supplemenarty Material 4.**

**Supplementary Material 5.**

**Supplementary Material 6.**

**Supplementary Material 7.**

## Data Availability

The raw sequence data reported in this paper have been deposited in the Genome Sequence Archive in National Genomics Data Center (GSA: CRA012308) that are publicly accessible at https://ngdc.cncb.ac.cn/gsa. The metadata for all samples included in this study is presented in Supplementary tables.

## Abbreviations

ALB: Albumin
ALT: Alanine aminotransferase
AMY-P: α-Amylase EPS pancreatic
AMY-L: α-Amylase EPS
A-CCP: Antibody to cyclic citrullinated peptide
A-HCV: Antibody to hepatitis C virus
A-TG: Antibody to thyroglobulin
A-TPO: Antibodies to thyroid peroxidase
APOA: Tina-quant Apolipoprotein A-1
APOB: Tina-quant Apolipoprotein B
AST: Aspartate aminotransferase
BILT: Bilirubin total DPD
CA125: Cancer antigen 125
CA153: Cancer antigen 15–3
CA199: Carbohydrate antigen 19–9
CA724: Cancer antigen 72–4
CEA: Carcinoembryonic antigen
CHE: Cholinesterase
CHO: Cholesterol
CK: Creatine kinase
CORT: Cortisol
CPEPTID: Connecting peptide
CREA: Creatinine
CRPHS: Cardiac C-reactive protein(latex) high sensitive
CYFRA: CYFRA 21–1
E2: Estradiol-E2
EPO: Huma erythropoietin
ET-1: Human endothelin 1
FERR: Ferritin
FPSA: Free prostate-specific antigen
FSH: Follicle-stimulating hormone
FT3: Free triiodothyronine
FT4: Free thyroxine
GGT: γ-Glutamyltransferase
GLU: Glucose
HDL: High-density lipoprotein
HE4: Human epididymal protein 4
IGE: Immunoglobulin E
IL6: Interleukin-6
LDIP: Lactate dehydrogenase
LDL: Low-density lipoprotein
LH: Luteinizing hormone
LIPC: Lipase
MYO: Myoglobin
NH3: Ammonia
PCT: Procalcitonin
PRL: Prolactin
ProBNP: N-terminal pro B-type natriuretic peptide
ProGRP: Progastrin-releasing peptide
T3: Triiodothyronine
T4: Thyroxine
TG: Thyroglobulin
TNT-HS: Troponin T hs (high sensitive)
TP: Total protein
tP1NP: Total procollagen type 1 amino-terminal propeptide
tPSA: Total prostate-specific antigen
TRIGL: Triglycerides
TSH: Thyrotropin
C3C: Tina-quant complement C3c
AFP: α-1-Fetoprotein
TESTO: Testosterone
PROG: Progesterone
PHO: Phosphate
