# Supplementary figures and images for: Longitudinal multi-omics analysis uncovers the altered landscape of gut microbiota and plasma metabolome in response to high altitude
Source: Microbiome. 2024 Apr 5;12:70. doi: 10.1186/s40168-024-01781-5 (PMC10996103; doi:10.1186/s40168-024-01781-5)

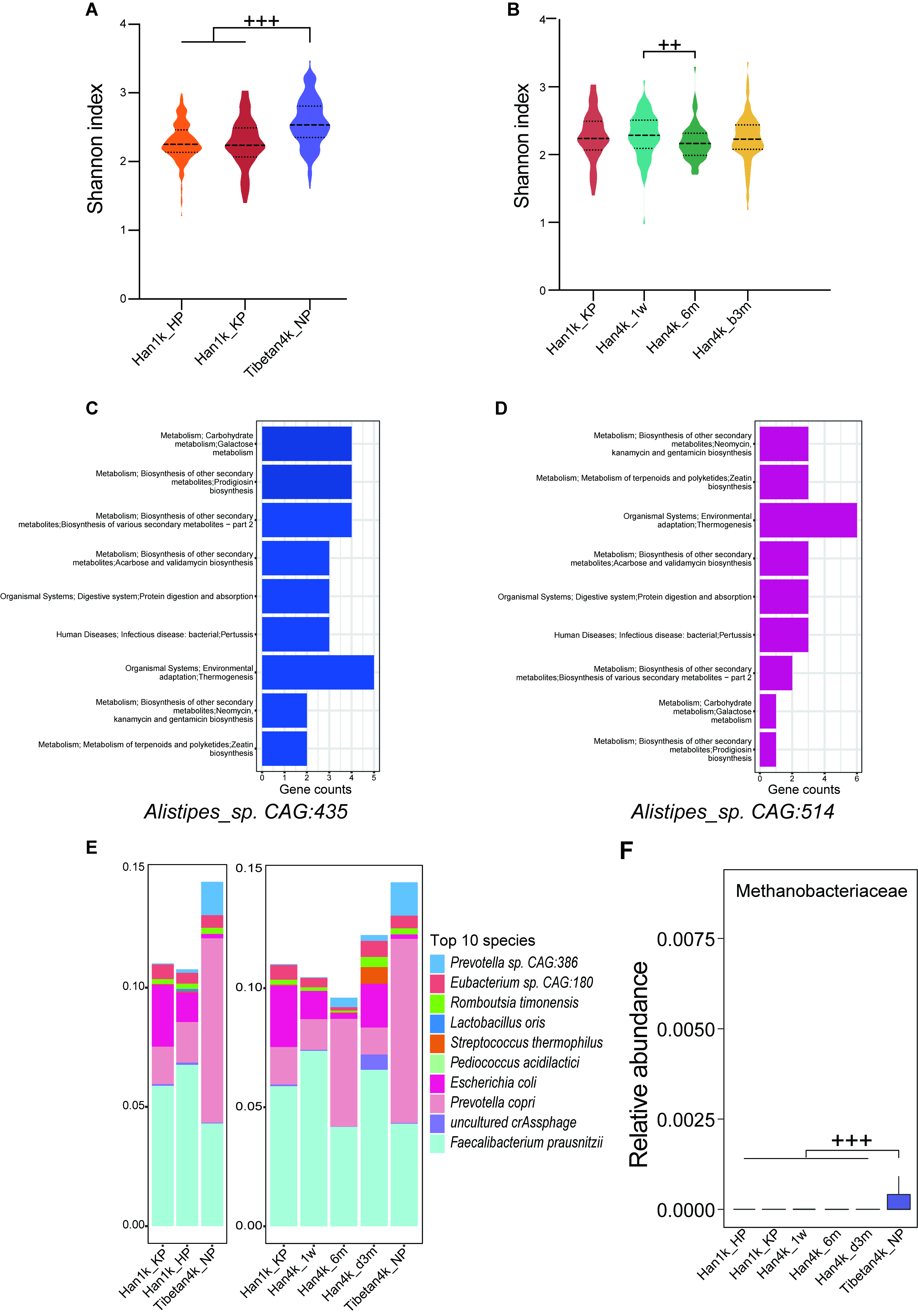

Supplement: Supplementary file 2 — Supplementary Material 1. [file 40168_2024_1781_MOESM1_ESM.tif]

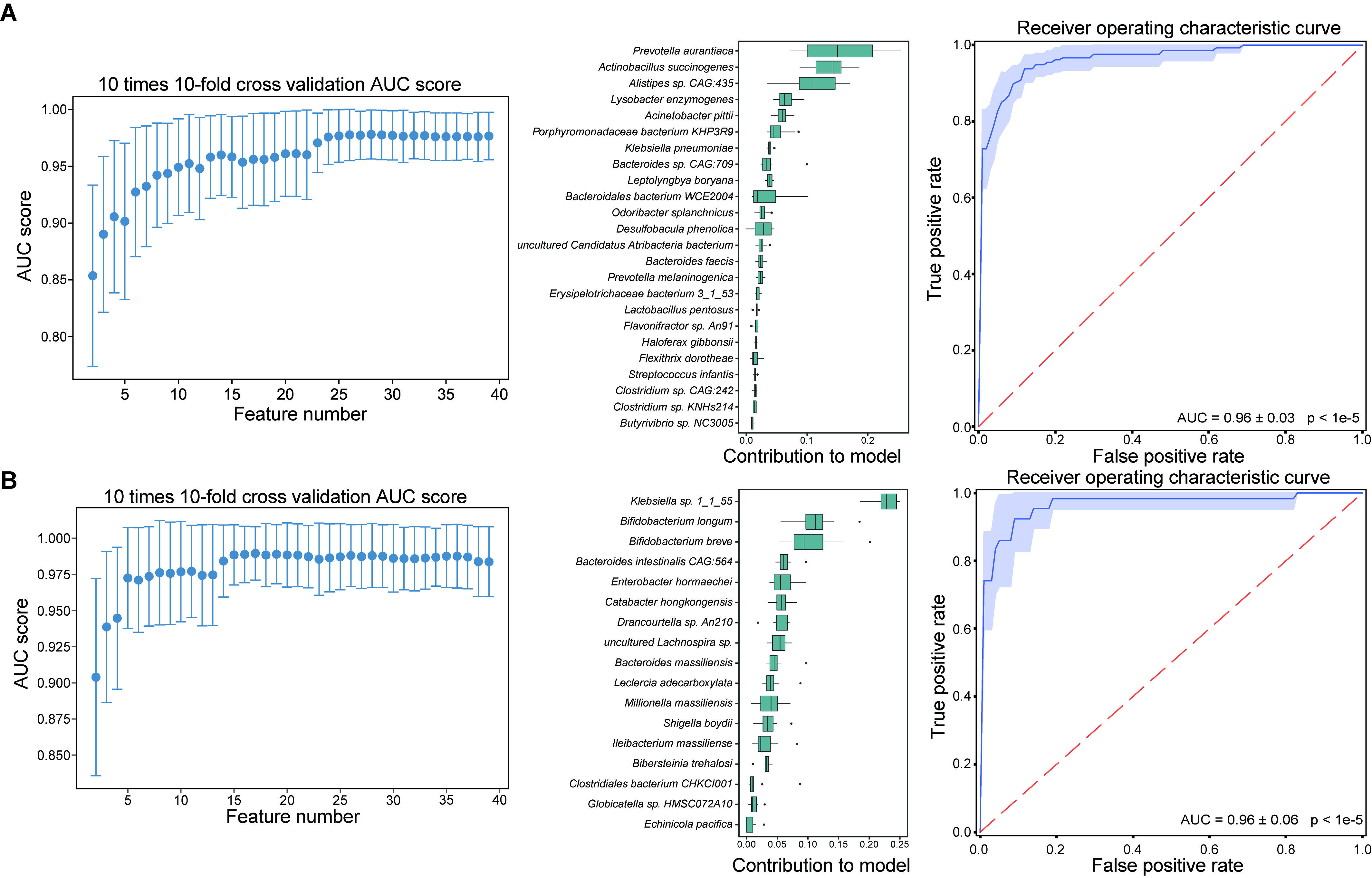

Supplement: Supplementary file 3 — Supplementary Material 2. [file 40168_2024_1781_MOESM2_ESM.tif]

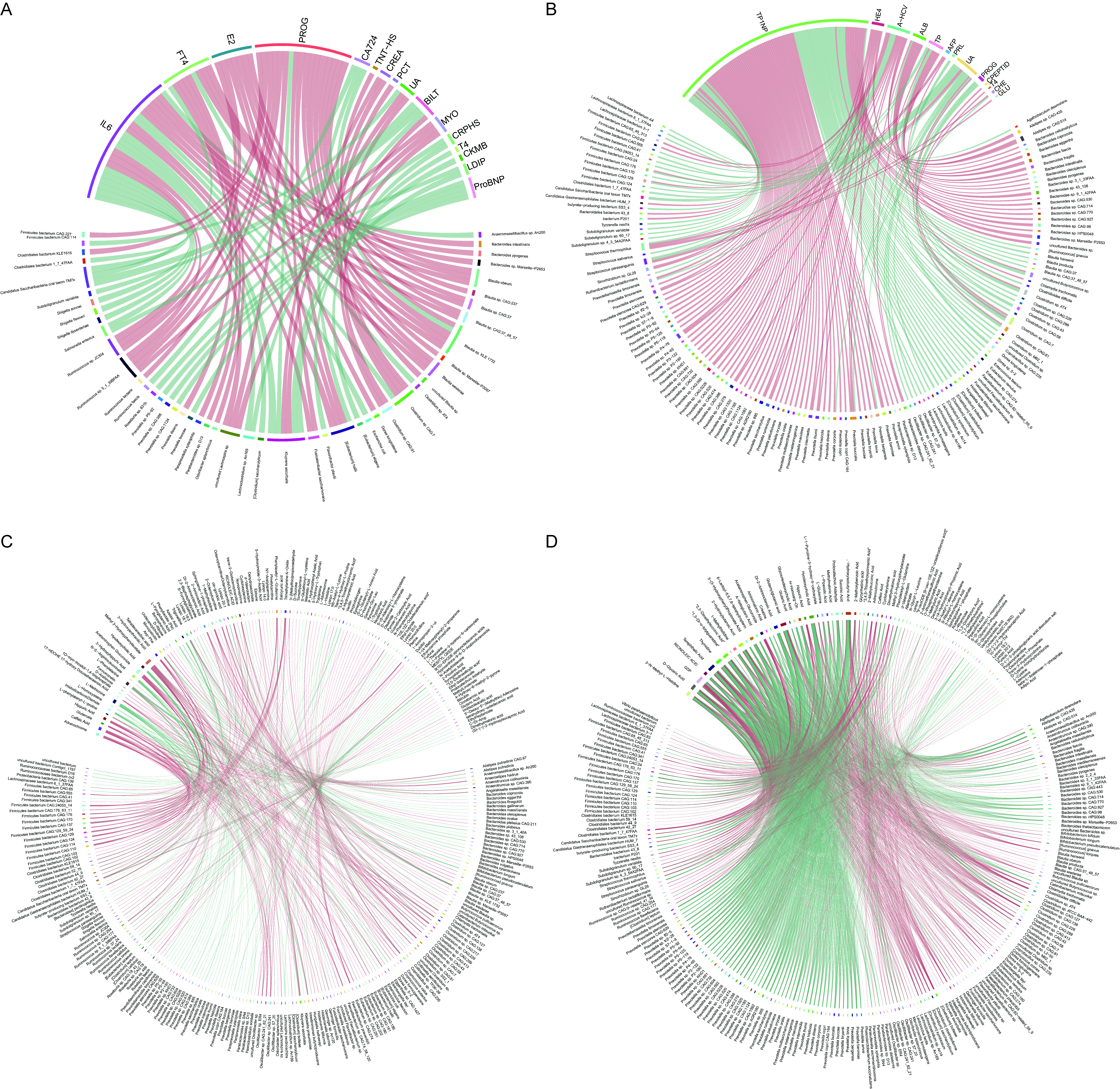

Supplement: Supplementary file 4 — Supplementary Material 3. [file 40168_2024_1781_MOESM3_ESM.tif]

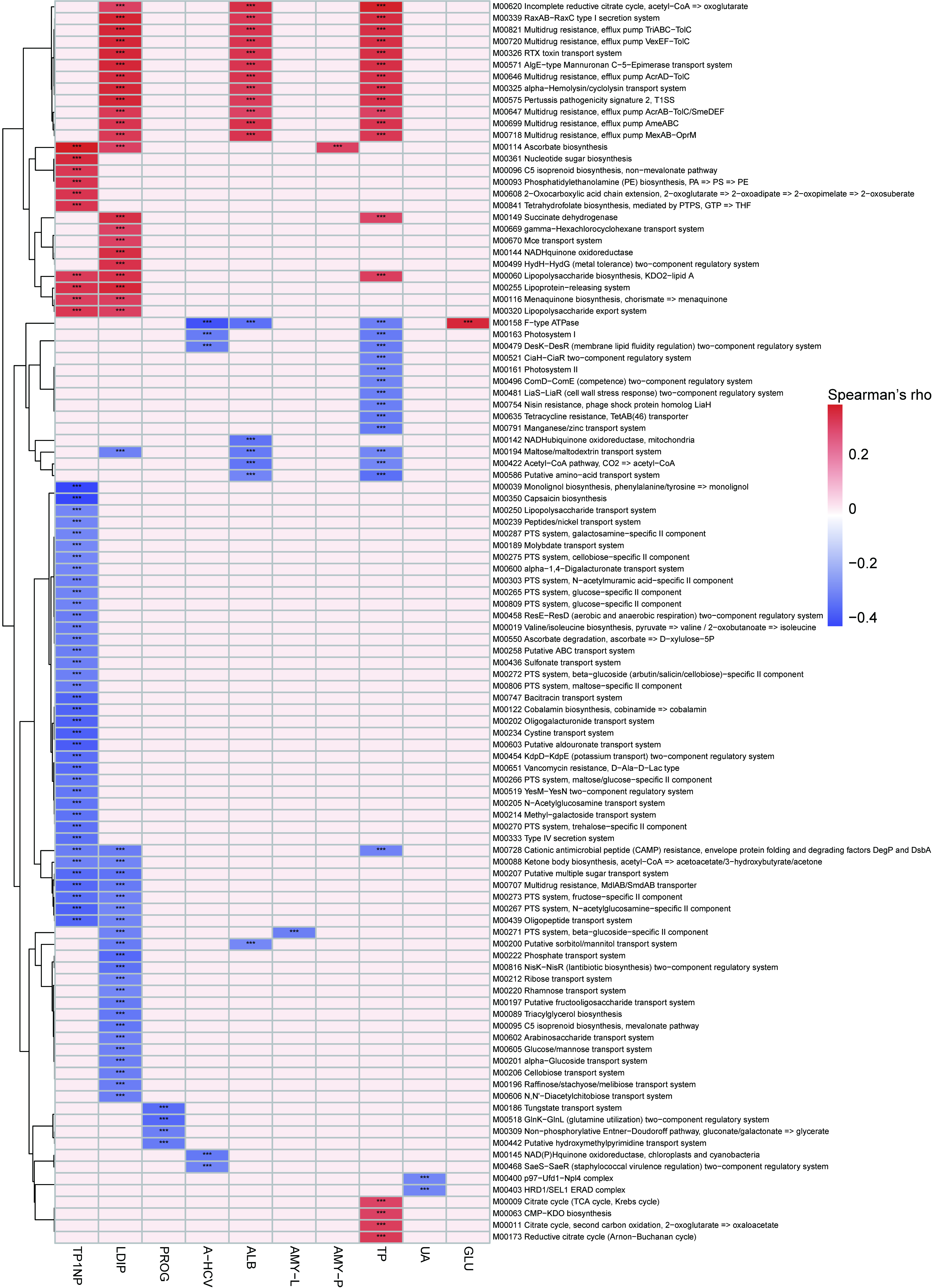

Supplement: Supplementary file 5 — Supplemenarty Material 4. [file 40168_2024_1781_MOESM4_ESM.tif]

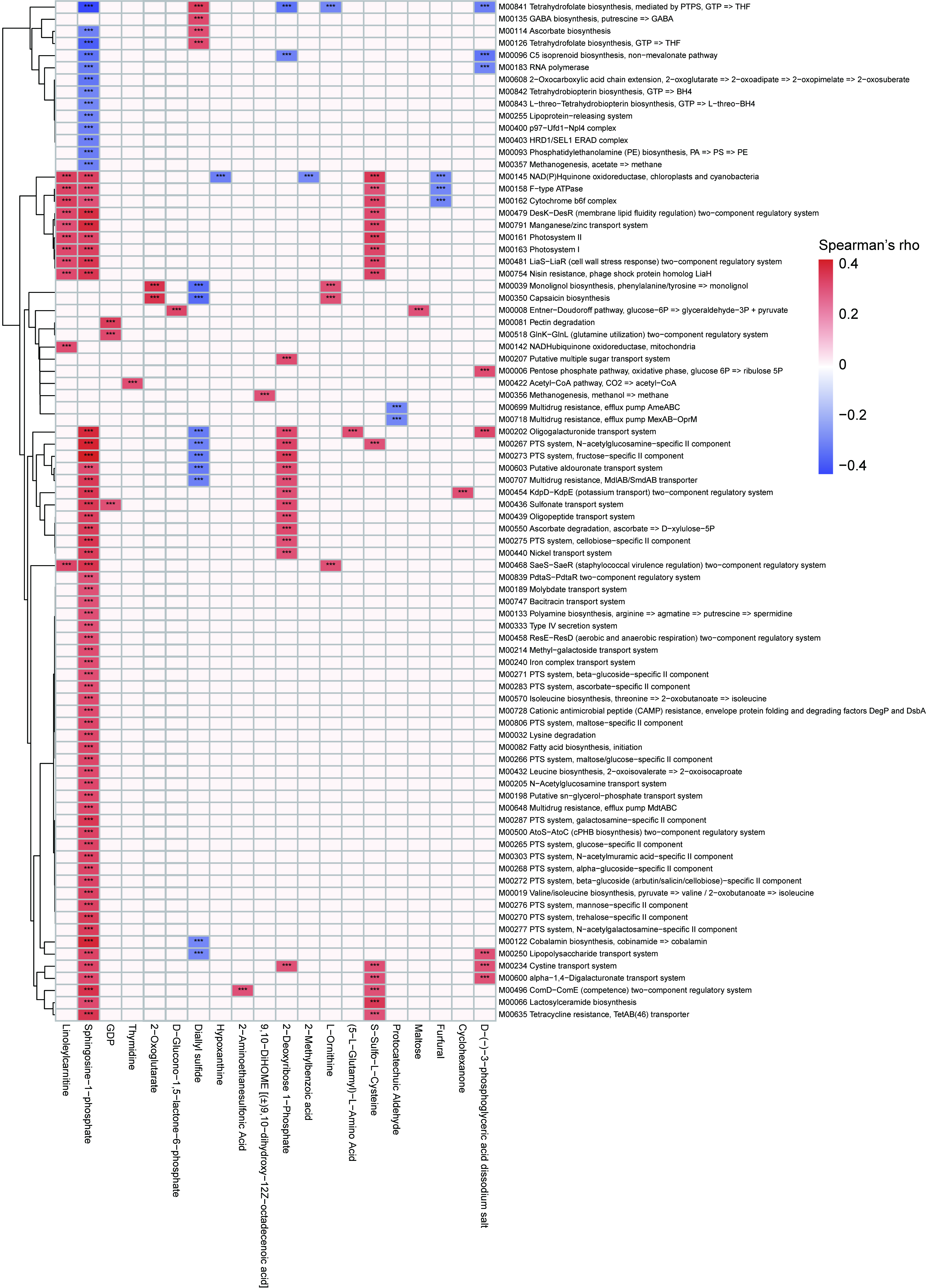

Supplement: Supplementary file 6 — Supplementary Material 5. [file 40168_2024_1781_MOESM5_ESM.tif]

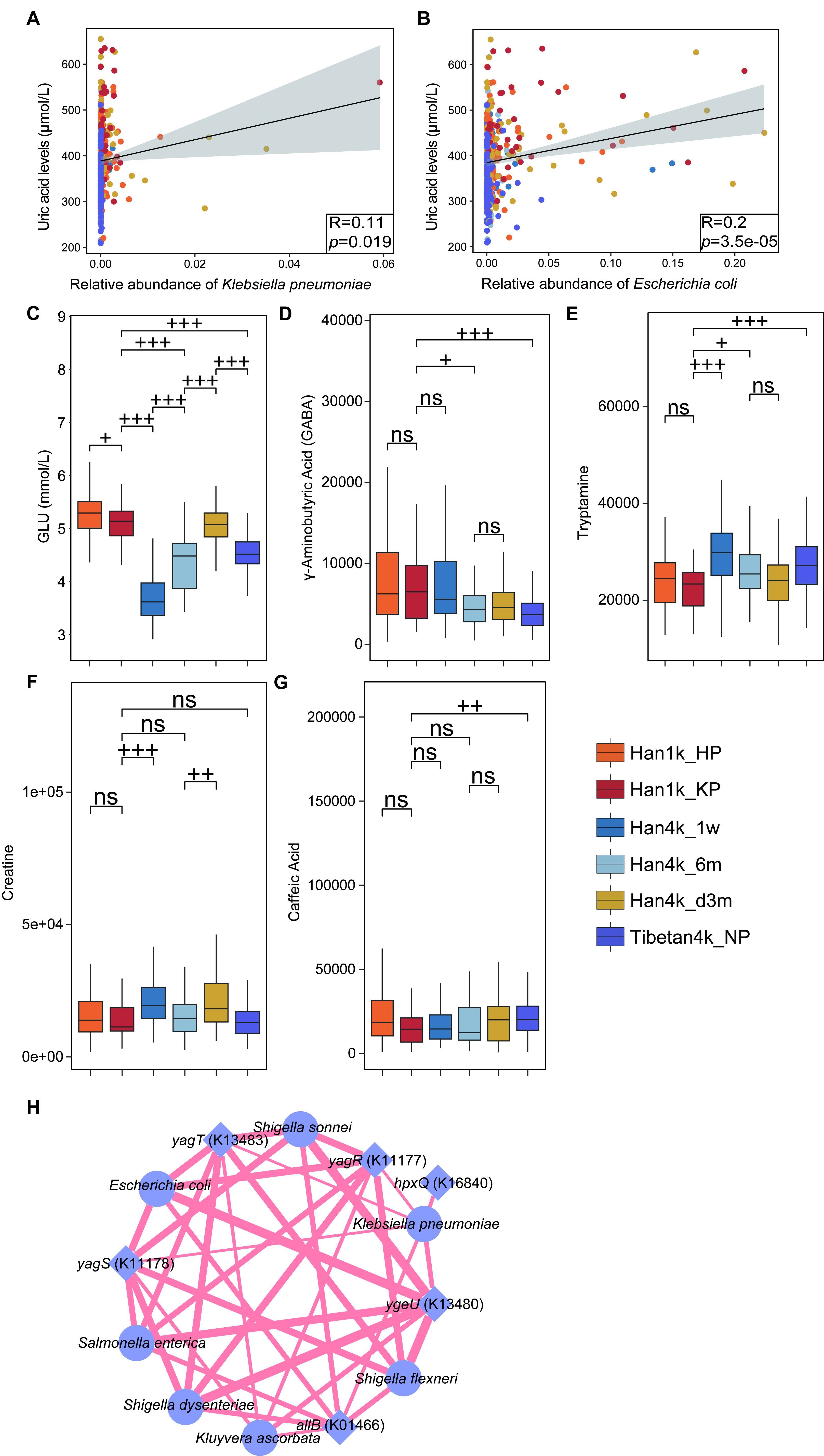

Supplement: Supplementary file 7 — Supplementary Material 6. [file 40168_2024_1781_MOESM6_ESM.tif]
